# Semi-field evaluation of a volatile transfluthrin-based intervention reveals efficacy as a spatial repellent and evidence of other modes of action

**DOI:** 10.1371/journal.pone.0285501

**Published:** 2023-05-11

**Authors:** Timothy A. Burton, Lewis Hambayi Kabinga, Limonty Simubali, Quinton Hayre, Sarah J. Moore, Jennifer C. Stevenson, Neil F. Lobo

**Affiliations:** 1 Eck Institute for Global Health, University of Notre Dame, Notre Dame, IN, United States of America; 2 Macha Research Trust, Choma, Choma District, Zambia; 3 Vector Control Product Testing Unit (VCPTU), Ifakara Health Institute, Environmental Health, and Ecological Sciences, Bagamoyo, Tanzania; 4 Vector Biology Unit, Department of Epidemiology and Public Health, Swiss Tropical & Public Health Institute, Basel, Switzerland; 5 Faculty of Science, University of Basel, Basel, Switzerland; 6 The Nelson Mandela African Institution of Science and Technology (NM-AIST), Tengeru, Arusha, Tanzania; 7 Department of Molecular Microbiology and Immunology, Bloomberg School of Public Health, Johns Hopkins University, Baltimore, MD, United States of America; University of Ghana Noguchi Memorial Institute for Medical Research, GHANA

## Abstract

Presently, the most common malaria control tools–i.e., long lasting insecticide-treated nets (LLINs) and indoor residual spraying (IRS)–are limited to targeting indoor biting and resting behaviors of *Anopheles* mosquito species. Few interventions are targeted towards malaria control in areas where transmission is driven or persists due to outdoor biting behaviors. This study investigated a volatile pyrethroid-based spatial repellent (VPSR) designed to bridge this gap and provide protection from mosquito bites in outdoor spaces. Southern Province, Zambia, is one such environment where outdoor biting is suspected to contribute to malaria transmission, where people are active in the evening in open-walled outdoor kitchens. This study assessed the VPSR in replica kitchens within a controlled semi-field environment. Endpoints included effects on mosquito host seeking, immediate and delayed mortality, deterrence, blood feeding inhibition, and fertility. Host-seeking was reduced by approximately 40% over the course of nightly releases in chambers containing VPSR devices. Mosquito behavior was not uniform throughout the night, and the modeled effect of the intervention was considerably higher when hourly catch rates were considered. These two observations highlight a limitation of this overnight semi-field design and consideration of mosquito circadian rhythms is recommended for future semi-field studies. Additionally, deterrence and immediate mortality were both observed in treatment chambers, with evidence of delayed mortality and a dose related response. These results demonstrate a primarily personal protective mode of action with possible positive and negative community effects. Further investigation into this primary mode of action will be conducted through a field trial of the same product in nearby communities.

## Introduction

Vector control measures have a large impact on malaria burden, accounting for an estimated 81% of total malaria reduction between 2000–2015 [1]. The existing interventions, long lasting insecticide-treated nets (LLINs) and indoor residual spraying (IRS), counter indoor late-night biting and indoor resting behaviors. These bionomic traits are generally common in many key *Anopheles* vectors of malaria including *An*. *gambiae s*.*l*. and *An*. *funestus s*.*l*., the primary vectors in much of sub-Saharan Africa. However, biting patterns have been observed to shift in response to interventions [2–4]. In addition, molecular identification has revealed unexpectedly high *Anopheles* species diversity in many settings demonstrating greater complexity in transmission dynamics [5–7]. These shifts in species compositions, densities, and bionomic traits are not well documented and may be highly spatially heterogenous, with one model estimating that Africans in general are experiencing 10% fewer of their mosquito bites while in bed or indoors in 2018 compared to 2003 [8]. In many settings, this shift in biting patterns has resulted in increased mosquito exposure outdoors when people are likely to be outdoors: generally, earlier in the evening and later in the morning [4, 9–14]. For vector control efforts, these behavioral shifts and other gaps in protection are identifiable in many settings and are becoming more relevant to malaria transmission in areas where much of the malaria burden has been reduced such as Zambia’s Southern Province where this study was conducted.

Community protection is a vital aspect of existing interventions including LLINs and IRS; by reducing local mosquito populations and therefore exposure of the community as a whole, the insecticidal action of these interventions can provide significant protection to a user’s unprotected neighbors [15]. This community effect somewhat depends on the resistance status of local mosquitoes and is a vital aspect of the continued effectiveness of these interventions [16–19]. While LLINs and IRS provide significant community effects through control of local malaria vectors, they both specifically target indoor biting and resting behaviors. Additional tools that target other mosquito behaviors may be required to provide personal and community protection in additional settings. There are no official recommendations for interventions to be deployed in these spaces, such as outdoors and in the early evening, where people may be at risk of mosquito biting [20]. Structural improvements, such as closing eaves and screening windows and doorways of homes, are possible alternatives to traditional indoor interventions but are impractical or inapplicable for many peridomestic and outdoor spaces [21]. Larval source management allows public health officials to reduce local mosquito populations but is impractical over large areas and does not directly target outdoor biting [20, 22].

Outdoor transmission is relevant in many low malaria settings, due in part to human behaviors in the early evening and morning which can expose them to mosquito activity [4, 23]. Outdoor human-mosquito interactions are quite variable, highlighting the advantages of flexibility, portability, and ease of use in interventions that target these behaviors. Volatile pyrethroid-based spatial repellent (VPSR) interventions incorporate these advantages and have shown promise in reducing mosquito landing behavior in prior studies [24–26], in addition to increasing mosquito mortality [27–29], and some evidence of reducing blood feeding behavior [27]. This study evaluates a volatile pyrethroid spatial repellent (VPSR) developed by Widder Bros., Inc. The pyrethroid active ingredient transfluthrin has been shown to cause mortality/knockdown effects in addition to repellency in *Anopheles* mosquitoes, including strains resistant to other pyrethroids [25, 30–34]. These outcomes are of epidemiological importance, with the undesirable possibility of repelled mosquitoes diverting to nearby hosts [35], and knockdown or kill effects possibly reducing transmission in a mechanism similar to the community effect provided by LLINs and IRS [36].

This study used a semi-field system at Macha Research Trust, southern Zambia, to evaluate the intervention utilizing entomological outcomes. The system is in a setting with seasonal and very low malaria transmission, with outdoor mosquito biting behavior and high ITN coverage. Much of the outdoor biting is thought to occur in the evenings and mornings while families are gathered in open-walled kitchen huts. The VPSR design could therefore reduce mosquito interactions in these spaces. The study design utilized the semi-field enclosure to conduct controlled release and recapture experiments to measure endpoints beyond mosquito landing. Study endpoints were designed to measure landing rate, repellency, and knock down during active use of the device, in addition to delayed effects on blood feeding, mortality, and fecundity after exposure. These study endpoints were employed to assess the efficacy and longevity of the VPSR devices.

## Materials and methods

### Ethical statement

This study was approved by the institutional review board at the University of Notre Dame (Protocol #: 18-05-4675) and by the local IRB at Macha Research Trust (IRB #: IRB0007649). Study participants were Macha Research Trust entomology staff who were fully informed of the risks and voluntarily provided informed oral consent. Their employment was not contingent on participation in the study. They did not receive additional compensation or incentives for the study but were paid at their normal pay rates for their work. Additional information regarding the ethical, cultural, and scientific considerations specific to inclusivity in global research is included in the Supporting Information (S1 Checklist).

### Intervention

**Fig 1:**
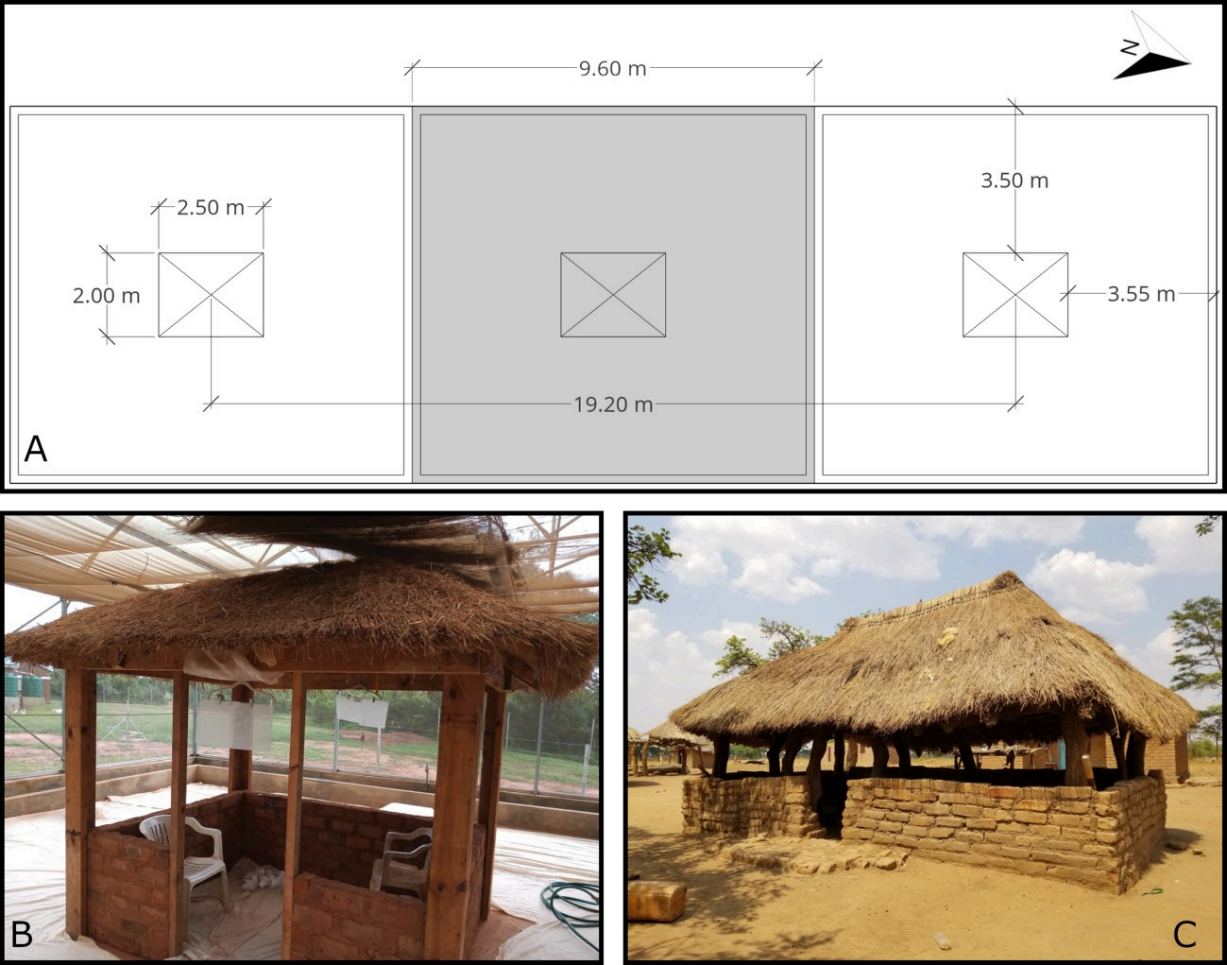
Structures and VPSR product deployment. (A) Floorplan of semi-field chambers used for nightly release-recapture experiments, generated using SketchUp for Web (© 2022, Trimble Inc.). Chamber one is located on the left (south), with chamber two on the right (north); the middle chamber was not used for this study. (B) VPSR devices deployed in a semi-field replica structure with the hut in an outdoor configuration (upper walls removed). (C) Example of a typical outdoor kitchen in the area.

The transfluthrin product tested is a volatile pyrethroid spatial repellent (VPSR) developed by Widder Bros., Inc. (Fig 1). Each device consists of two 25x25cm sheets and serves as a passive emanator of the volatile pyrethroid transfluthrin to provide an area of protection from mosquito activity for up to a month or longer without replacement. The devices were deployed within the semi-field kitchen structures by hanging them under two opposite eaves of the structure, 1.5 meters from the floor (Fig 1). Two devices were deployed to each kitchen (one per side).

### Semi-field system

The semi-field system is a large enclosure walled with a fine mesh to prevent the ingress of local insects or the egress of test mosquitoes whilst allowing temperatures to largely equilibrate with the external environment (Fig 1). The two test chambers are separated by an unused third test chamber, and each chamber is isolated from the other by interior walls made of the same fine mesh material. Each chamber measures approximately 10m by 10m, with a lower ceiling of fine mesh about 3m above the ground. The entire enclosure sits under a simple sealed plastic roof for protection from the elements. Each chamber is surrounded by a narrow ditch filled with water and a mild surfactant, which prevents crawling insects that might prey on mosquitoes from reaching the interior of the chambers. During the experiments, the cement floors of the chambers were covered in white cloth which was wetted with water before each experiment. These cloths served to increase the relative humidity within chambers while providing a backdrop to easily find dead mosquitoes. This design allowed for the simultaneous evaluation of the intervention against a baseline control using mosquitoes reared from the same generation in an insectary.

The test huts located in each chamber represent shelters used in the area. For this study, the upper half of test shelter walls were removed to replicate the design of local kitchen shelters (Fig 1). These shelters have 2m x 2.5m floors, with brick walls roughly 1m tall except in the doorway, an additional 1m of open sides, and a grass roof.

### Study design

This experiment used a simple 2x2 Latin square rotational design between the test chamber and control chamber (negative control) to account for chamber and weekday effects. The human collectors stayed in the same chamber on each night to enable the collector and chamber effects to be coupled as a single source of bias. Experiments were conducted every third or fourth night (on Monday and Thursday nights) to provide a wash out period between replicates and allow the transfluthrin and any host associated odors to dissipate between rotations.

Experiments were conducted from December 2019 through April 2020. Temperature and humidity were recorded at 5-minute intervals for the duration of experimental nights using a data logger (Onset HOBO). External rainfall and moon phase were recorded categorically for each night. Experiments took place over 32 nights, including two nights of baseline collection with no devices in place. Ten nights were dedicated to testing ten separate fresh sets of VPSR devices. Four of these sets were tested weekly for five weeks over the remaining twenty nights. Between timepoints, these devices were kept freely hanging out of direct sunlight above an open office window.

#### Mosquitoes and insectary conditions

Laboratory-reared *Anopheles gambiae s*.*s*. (Kisumu strain) were used for all experiments. This colony is well established at MRT and is susceptible to all pyrethroid insecticides (unpublished data). Mosquitoes are reared in large cages and experimental groups were kept in 30cm x 30cm BugDorm cages. Reared mosquitoes were provided with 10% sucrose solution *ad libitum*. These cages were held in the insectary at 27 degrees C and 80% humidity until transfer to the experimental chambers. Approximately 250–300 2–5-day old female mosquitoes were selected for release into per experimental chamber on a given night. Sucrose was removed from cages to starve all experimental mosquitoes four hours prior to their release in experimental chambers.

#### Cage setup

**Fig 2:**
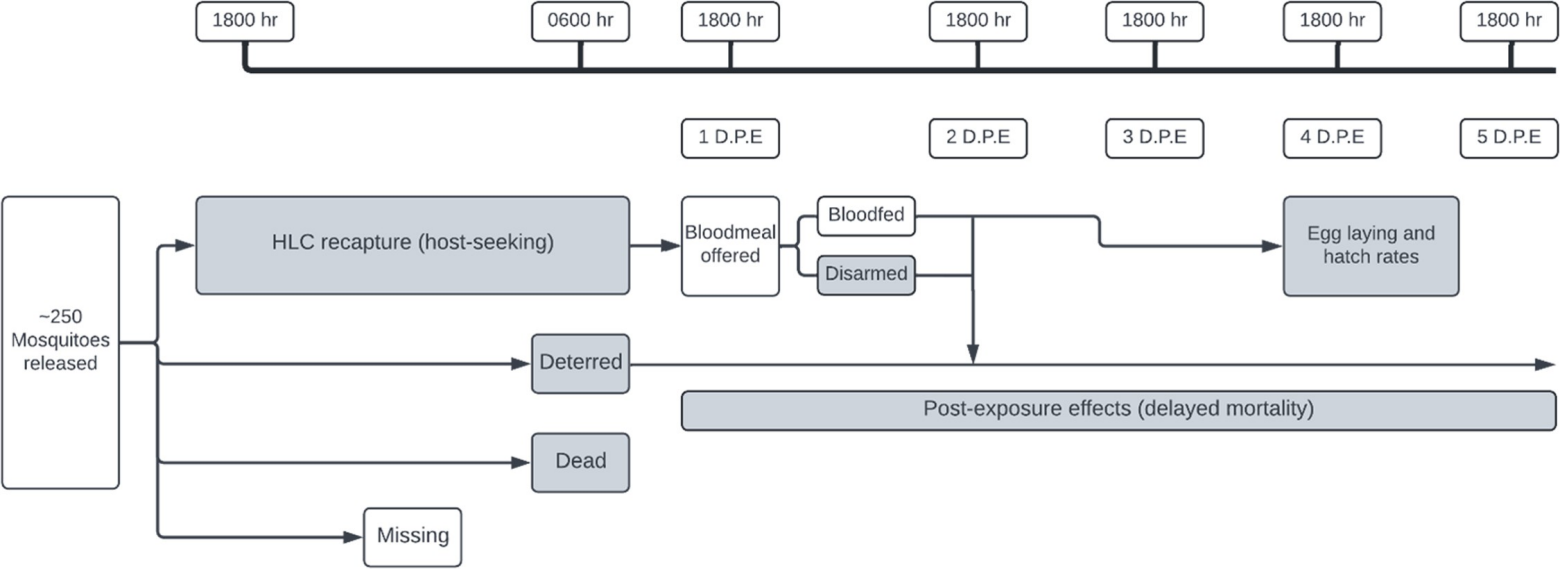
Timeline of experimental replicates. Experimental endpoints are indicated in gray boxes. Bloodmeals were offered to host-seeking mosquitoes at 1-day post-exposure, 24 hours after mosquitoes were released into experimental chambers. Egg-laying rates were measured overnight after 4-days post-exposure, with hatch rates measured the following morning. Delayed mortality was noted for all days starting at 1-day post-exposure for deterred and host-seeking mosquitoes.

Mosquitoes were selected in the early afternoon and placed into separate cages for each chamber before experiments commenced. VPSR devices were deployed to the appropriate chamber before 17:00, while the opposite chamber served as a no-device control. At 17:00, the chambers were prepared for the nightly replicate. This included filling the perimeter troughs with water and a mild surfactant and laying out and wetting white cloth on the floors of each chamber. At 17:30, mosquitoes were moved to the experimental chambers from the insectary and released from their cages to acclimate in chambers. Collectors entered chambers at 18:00, signaling the start of an experimental replicate (Fig 2).

### Endpoints

Table 1.

**Table 1:** Definitions of study endpoints.

Study Endpoints		
**Endpoint**	Definition	Model output
**Landing/Host-seeking**	Mosquito exhibits host-seeking behavior and lands on the mosquito collector	Risk Ratio
**Disarming**	Blood feeding rate of host-seeking mosquitoes	Odds Ratio
**Fecundity**	Egg laying and hatch rates of fed mosquitoes	Risk Ratio
**Delayed Mortality** **(Landing population)**	Landing mosquito death up to five days after exposure	Odds Ratio
**Deterrence**	Mosquito does not exhibit host-seeking behavior overnight, recovered alive in the morning	Risk Ratio
**Delayed Mortality** **(Deterred population)**	Deterred mosquito death up to five days after exposure	Odds Ratio
**Knockdown/mortality**	Mosquito does not exhibit host-seeking behavior overnight, recovered dead in the morning	Risk Ratio

#### Host-seeking behavior

Mosquitoes were recaptured from within the shelters by trained entomologists performing human landing collection (HLC). This is the gold standard for mosquito collection, where collectors use a mouth aspirator to collect mosquitoes that land on them [37]. Collections took place overnight, with mosquitoes collected and counted separately by hour from 18:00–06:00. Collectors were provided coffee and listened to music or radio to aid in staying awake. All mosquitoes caught by HLC were moved to the insectary at the end of each hour for additional experiments. Host seeking behavior measured by HLC constituted the primary experimental endpoint and was calculated per night by dividing the total HLC recapture in a chamber by the number of mosquitoes released. Hourly host seeking behavior was calculated by dividing the HLC recapture for a single hour by the number of mosquitoes remaining in the chamber at the start of that hour.

#### Deterrence and mosquito knockdown/mortality

Following HLC collections, additional collectors entered each chamber at 08:00 and collected all remaining alive and dead mosquitoes. Two collectors per chamber actively searched for remaining mosquitoes using mouth aspirators, with one searching inside the hut and the other the remaining portions of the chamber including the perimeter ditch. Collectors rotated between chambers halfway through at 09:00, finishing clearing chambers at 10:00. The locations of these mosquitoes were noted as inside/outside the shelter, with survivors moved to the insectary and knocked down mosquitoes counted and discarded. Deterrence was informed by the proportion of released mosquitoes found alive in these morning collections that were not captured in HLCs (they were deterred from landing on HLC collectors). Knocked down or dead mosquitoes were not monitored for recovery, and direct mosquito mortality was calculated as the proportion of released mosquitoes found knocked down or dead.

#### Overall recovery rate

The overall recovery rate from chambers was included as a general measure of experimental bias and to detect effects which were not captured by study endpoints. The overall recovery rate was calculated by dividing the sum of mosquitoes recovered from the chamber (host-seeking, deterred, and knocked down) by the number of mosquitoes released into the chamber the previous night. On a small number of experimental nights (n = 5/64), this recovery rate was slightly higher than 100%, possibly due to miscounting or mosquito survival inside the chambers between replicates. For these chambers, the number of released mosquitoes was amended to yield a recovery rate of 100%.

#### Delayed effects

In the insectary, HLC-captured and deterred mosquitoes were separately followed for five days post-exposure (d.p.e.) to measure delayed mortality effects of transfluthrin exposure. Additionally, host seeking (HLC captured) mosquitoes were offered a bloodmeal from an anesthetized mouse at roughly 18:00, or 12-24-hours post-capture to measure inhibition of blood feeding behavior (disarming). These mosquitoes were sugar-starved for four hours prior to the bloodmeal. The numbers of bloodfed females were counted and provided wetted filter papers for egg laying three days later (Fig 2). The numbers of laid eggs were counted and deposited into fresh larval pans; the hatched larvae were counted the next day and discarded. These data were used to post-exposure blood feeding, egg laying (fecundity), and egg hatch rates (fertility).

### Statistical analysis

Generalized linear mixed models with an appropriate error distribution and a log link function were used for analysis. Host seeking, knockdown, and deterrence were analyzed using a Poisson distribution, which included an offset term to adjust for the number of mosquitoes exposed to the outcome (e.g., released into each chamber). Fixed effects included treatment and the age (in weeks after opening) of the VPSR device, chamber, temperature, and humidity with the date of experiment included as a random effect to account for day-to-day variation in mosquito behavior and other unmeasured factors contributing to experimental variation. An interaction between treatment and device age was added to measure the effect of time on efficacy. Model coefficients were exponentiated and reported as rate ratios with the control set as the reference. The remaining endpoints gathered in the insectary (blood feeding, delayed mortality, fecundity) were modeled using a binomial distribution with the addition of cage density as a fixed effect. Models were evaluated and selected based on Akaike information criterion (AIC), with some fixed effects dropped from specific models when their addition reduced model fit. All data analysis was conducted in R version 4.0.2. Data was cleaned, summarized, and plotted using the tidyverse packages ‘tidyr’, ‘dplyr’, and ‘ggplot2’. Generalized linear models were generated and analyzed with the ‘lme4’ and ‘arm’ packages.

## Results

Over the course of data collection from December 2019 through April 2020, the mean temperature during the hours of experiments was 21 degrees C (s.d. = 1.6), decreasing slightly over time. Mean nightly humidity was 83.9% (s.d. = 13.5%), only dipping below 75% for a few nights in December and early 2020. In general, the early period had warmer and sometimes dryer nights (S1 Fig).

### Recovery of released mosquitoes and sources of bias

A total of 8885 mosquitoes were released across 34 nights (261/night) in untreated chambers, compared to 7848 across 30 nights (262/night) in treatment chambers. The additional four nights represent a baseline period which was not included in comparison models. An identical number of mosquitoes were released into both chambers on a given night. The overall recovery rate, defined as the number of mosquitoes that were recovered from the chambers by all experimental endpoints, was similar between treatment (84.9%) and control (89.7%) (RR: 0.95 [0.90–1.00], p = 0.061), as well as between chambers (86.4% vs 88.5%; (RR: 1.02 [0.99–1.06], p = 0.25). There was no observed significant effect of treatment, chamber/volunteer, or any other predictors on the recovery rate (full model in S1 Table).

### Host-seeking behavior of mosquitoes–protective efficacy

**Fig 3:**
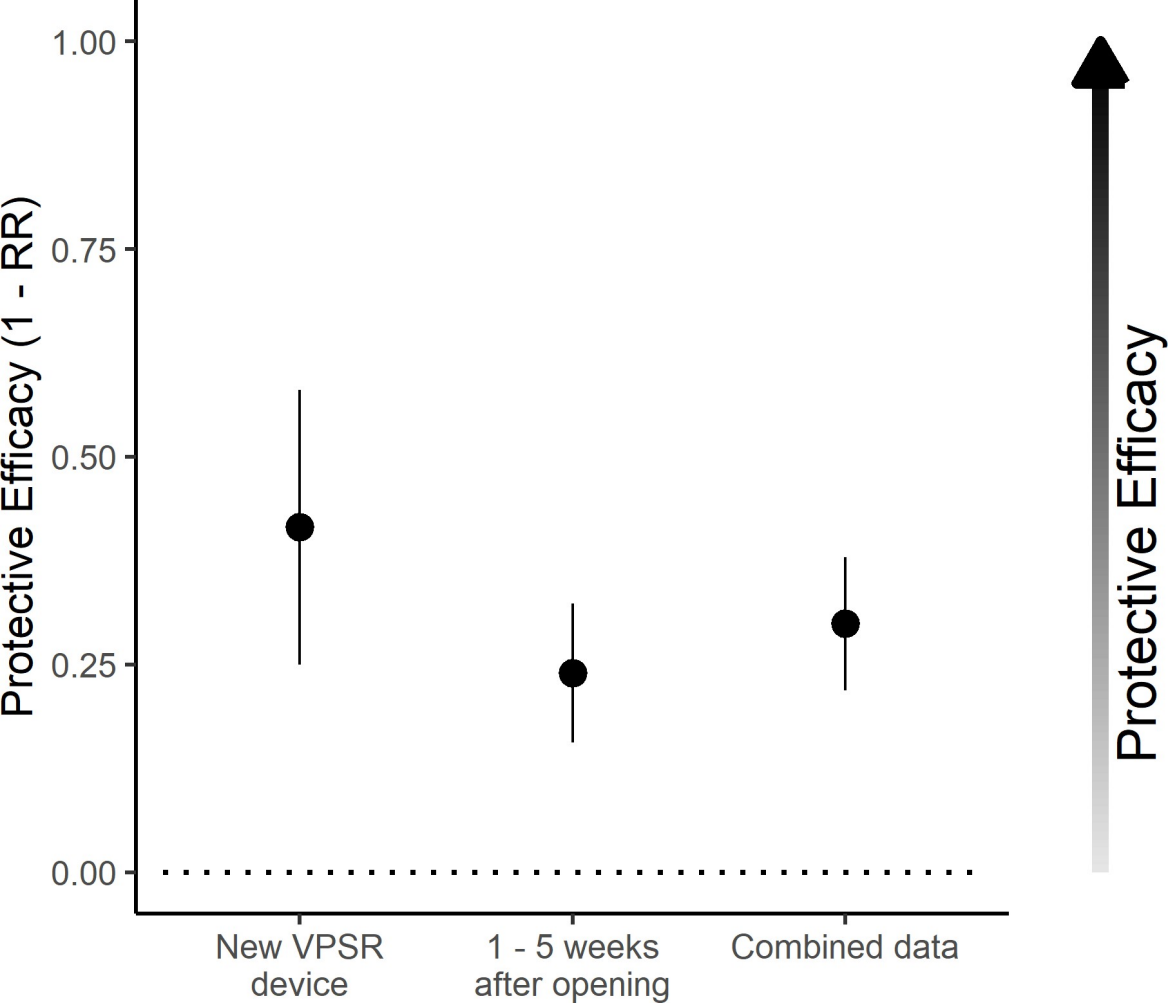
Protective efficacy during overnight human landing in treatment chambers relative to control chambers. The Y axis displays the risk ratio associated with VPSR device exposure, calculated by dividing the proportion of mosquitoes captured through the night in treatment chambers by the corresponding proportion in control chambers. The calculated RR has been subtracted from 1 to display protective efficacy as increasing along the Y-axis. Results are separated by age category of the treatment device on the X axis. The dotted horizontal line refers to a ratio of 1 (no change between groups) with increasing values on the Y axis referring to increased protective efficacy.

**Fig 4:**
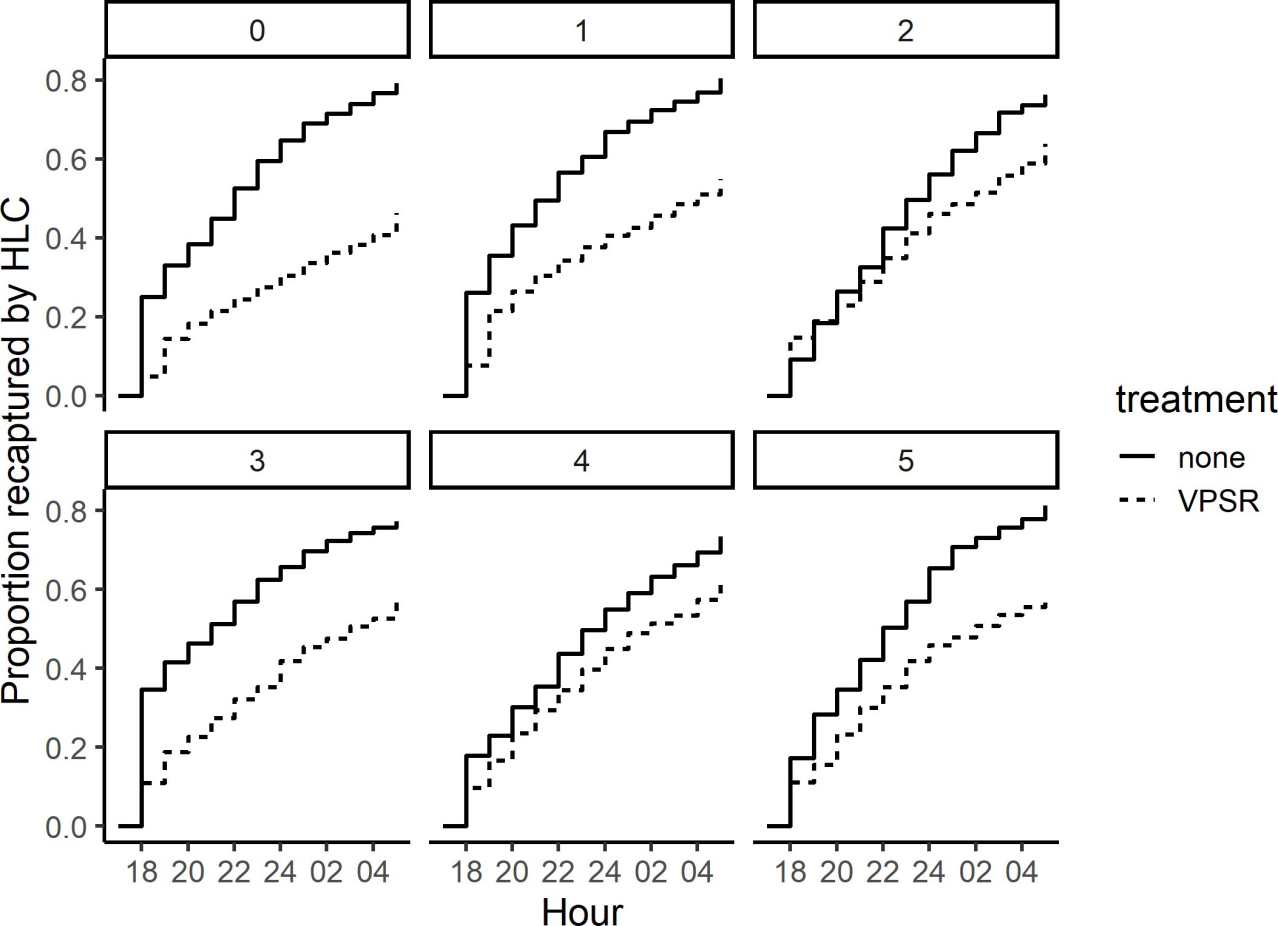
Cumulative overnight proportion of recapture by human landing collection in semi-field experiments of released mosquitoes in treatment and control chambers. The proportion of mosquitoes recaptured in treatment and control chambers is displayed on the Y axis cumulatively by hour along the X axis. Each panel represents experimental nights with the corresponding device age in weeks, up to five weeks past opening.

In total, 7033/8885 (79.2%) of mosquitoes released in control chambers were captured by HLC, compared to 4319/7848 (55.0%) in treatment chambers. The greatest protective efficacy (PE) was observed using fresh devices, with PE remaining but generally decreasing through the five tested weeks (Figs 3 and 4). The treatment was associated with reduced mosquito landings across all timepoints when analyzed by night (RR: 0.61 [0.57–0.65], p < 0.001), or by hour (RR: 0.37 [0.34–0.40], p < 0.001). Overall, mosquito host-seeking across all chambers decreased later in the night (hourly RR: 0.95 [0.95–0.96], p < 0.001). The reduction of host seeking associated with treatment declined slightly as experiments progressed into the night (hourly RR: 1.02 [1.01–1.03], p = 0.003). Both analyses showed a decline in efficacy in the weeks after opening (weekly “device age” RR: 1.07 [1.05–1.10], p < 0.001 based on the all-night model; RR: 1.13 [1.10–1.16], p < 0.001 based on the hourly model).

Nightly temperature and humidity had no effect on control HLC recapture rates, but both were associated with reduced overnight VPSR treatment efficacy (scaled temperature RR: 1.10 [1.04–1.17], p < 0.001; scaled humidity RR: 1.06 [1.00–1.12], p = 0.04). This is likely because higher scaled hourly humidity was associated with greater overall mosquito activity (RR: 1.11 [1.02–1.21], p = 0.01). Higher hourly temperature was associated with reduced hourly treatmente efficacy (scaled temperature RR: 1.08 [1.03–1.12], p < 0.001), while higher hourly humidity was associated with increased efficacy (scaled humidity RR: 0.93 [0.89–0.97], p < 0.001). Chamber had no overall effect on human landing rate. Full model coefficients are provided in S1 Table.

### Deterrence

In total, 21.9% (n = 1717/7848) of mosquitoes released in test chambers were found alive the next morning, compared to 7.7% (n = 680/8885) of mosquitoes in control chambers (RR: 3.83 [3.30–4.44], p < 0.001). This deterrence effect declined with weekly device age (per-week RR: 0.86 [0.81–0.91], p < 0.001) (S1 Table). Of these surviving mosquitoes, 76.9% (n = 1321/1717) were found outside of the kitchen structure in test chambers compared to 60.4% (n = 411/680) in control chambers.

### Knockdown

**Fig 5:**
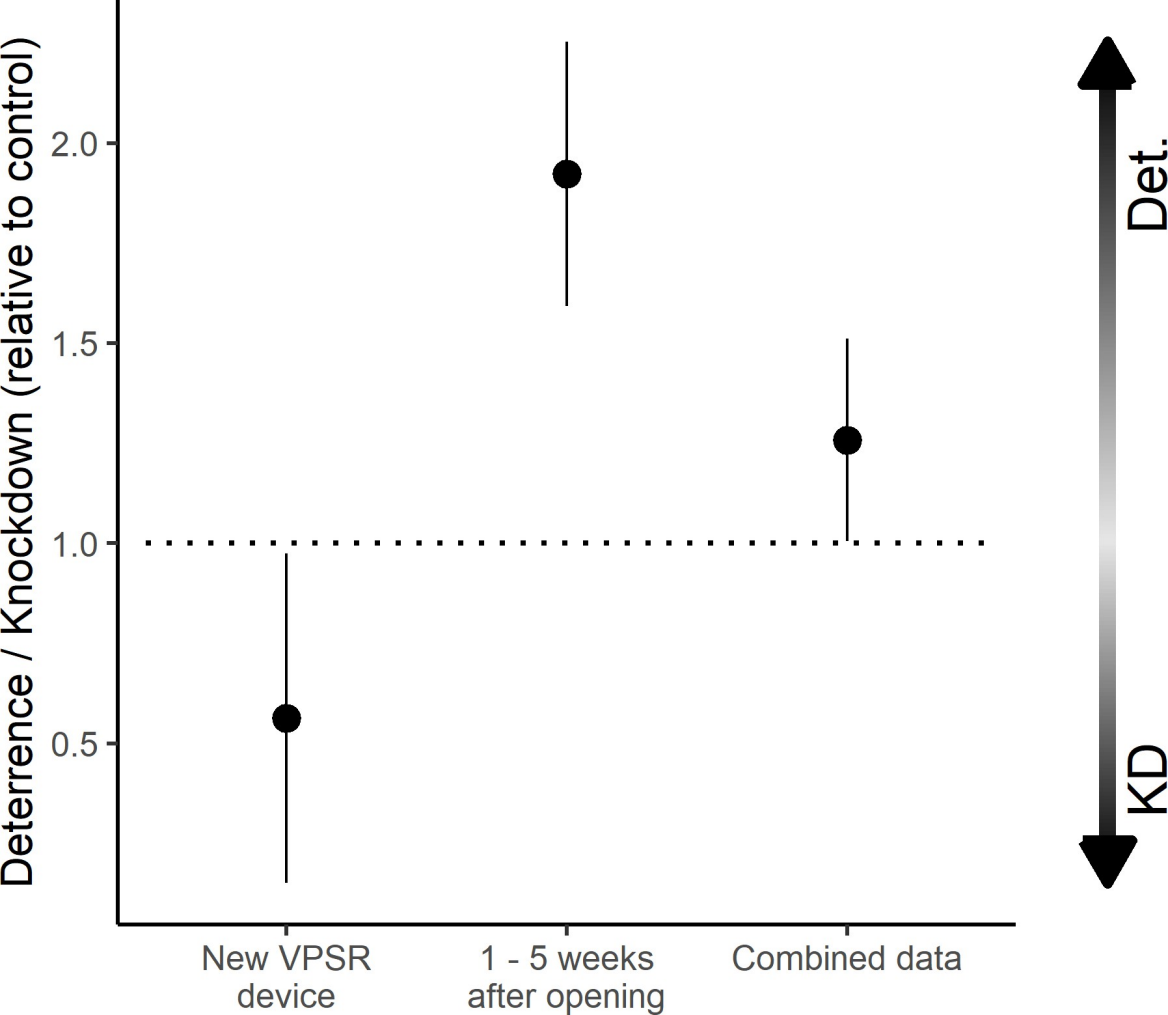
Ratio of deterrence compared to mortality associated with VPSR exposure in the semi-field system. The ratio of deterrence (captured alive outdoors and abbreviated to “Det.”) between treatment and control chambers was divided by the ratio of knockdown (KD) between treament and control chambers and plotted on the y axis. Results were separated by age category on the x axis. The dotted horizontal line refers to a ratio of 1 (no change between groups). Ratios greater than one indicate higher deterrence relative to mortality.

Mosquito knock-down was also elevated in treatment chambers (8.0% of all released mosquitoes, n = 626/7848)) compared to control chambers (2.9%, n = 261/8885). The largest difference was observed using freshly opened VPSR devices (15.5% in test chambers vs 2.3% in control), and these effects subsided in the five weeks after opening. Overall, there was no observable chamber effect on knock-down (6.1% vs 6.0% between chambers for all experimental nights). The treatment was associated with a substantial increase in the proportion of mosquitoes knocked down (RR: 5.88 [4.50–7.68], p < 0.001), with the effect decreasing with device age (weekly “device age” RR: 0.71 [0.65–0.78], p < 0.001) (S1 Table). The ratio of deterrence relative to knockdown increased with weekly device age, with fresh devices resulting in higher knockdown relative to deterrence compared to older devices, which trend towards heightened deterrence overall (Fig 5).

### Post-exposure survival of host-seeking and deterred mosquitoes

The survival of mosquitoes recaptured while host-seeking (HLC captured) and moving away from the VPSR treatment (deterred) was observed separately for five days post-exposure (d.p.e). Overall mortality of control mosquitoes was 4.7% (n = 319/6846) at one d.p.e. and 22.5% (n = 1543/6846) at five d.p.e. This was slightly elevated among treatment exposed mosquitoes at one d.p.e, 6.5% (n = 391/6036) and five d.p.e. 30.7% (n = 1856/6036).

**Fig 6:**
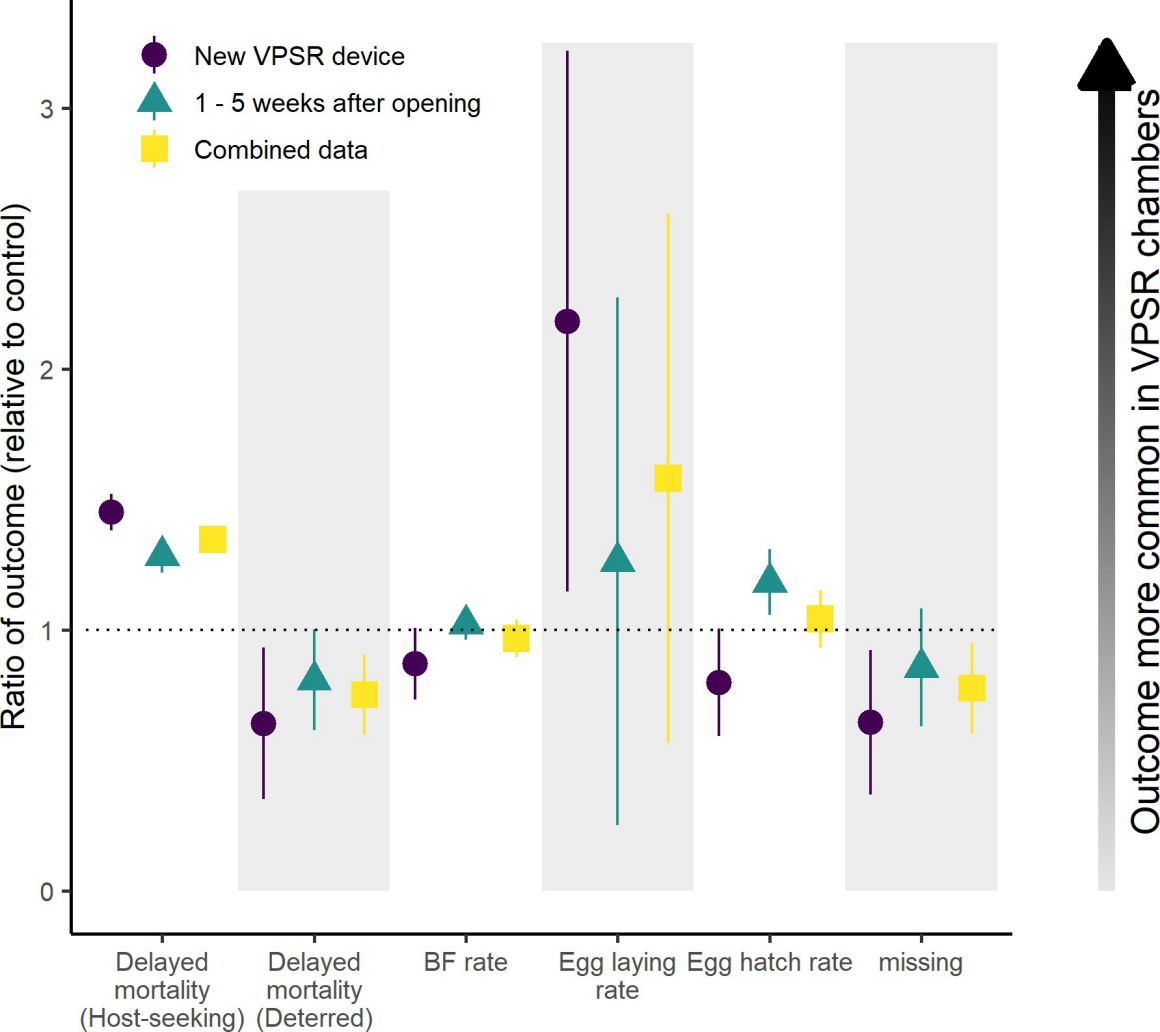
Risk ratios of secondary outcomes to VPSR exposure. Y axes represent the risk ratio of each outcome for intervention exposed mosquitoes compared to control chambers. Ratios were calculated based on the age category of the VPSR devices separated on the X axis by color and symbol and denoted in the figure legend. The dotted horizontal line represents risk ratio of 1, or no change between exposure and control. Numbers greater than one indicate outcomes which are more common in treatment chambers compared to controls, while numbers lower than one indicate outcomes which were lessened in treatment chambers.

Among host-seeking mosquitoes, control mortality was 4.4% at one d.p.e. (n = 273/6201) and 18.6% at five d.p.e. (n = 1155/6201) and was again slightly higher in the treatment exposed mosquitoes: 6.1% (n = 262/4319) and 24.6% (n = 1061/4319), respectively. Conversely, mortality of deterred mosquitoes in control chambers was 7.1% after one d.p.e. (n = 46/645) followed by 60.2% at five d.p.e. (n = 388/645), compared to 7.5% (n = 129/1717) and 46.3% (n = 795/1717) at each time point in treatment chambers (Fig 6).

VPSR exposure was associated with increased mortality at one day (OR: 2.42 [1.43–4.09, p = 0.001) and five d.p.e. (OR: 1.50 [1.15–1.96], p = 0.003) compared to controls. Higher temperature during the night of capture was associated with slightly higher mortality after one day (scaled temperature OR: 1.25 [1.01–1.55], p = 0.043) and five days (OR: 1.17 [1.02–1.33], p = 0.025). The deterred population of mosquitoes experienced similar mortality compared to the host-seeking population after 24 hours and increased mortality at five d.p.e. (OR: 4.54 [1.58–13.02], p = 0.005). The interaction between population and treatment was borderline significant after five days (OR: 0.34 [0.11–1.01], p = 0.053). This interaction term can be interpreted as the effect of the VPSR device on mortality of deterred mosquitoes relative to the overall effect of the device on all mosquito delayed mortality. Caged mosquito density was included as a predictor in all models, and density was not significantly association with decreased mortality at 5 d.p.e. (OR: 0.83 [0.69–1.01], p = 0.057). The cage density significantly interacted with treatment-, and higher cage densities were associated with decreased mortality at 1 d.p.e. (OR: 0.38 [0.23–0.64], p < 0.001) and 5 d.p.e. (OR: 0.69 [0.52–0.90], p = 0.007). Full model coefficients are provided in S2 Table.

### Blood feeding behavior of host-seeking mosquitoes

Blood feeding rates of host-seeking mosquitoes were measured 12–24 hours post exposure and were slightly lower in VPSR exposed mosquitoes during experimental nights which used freshly opened devices (83.0% compared to 95.8% in control mosquitoes; Fig 6). Blood feeding rates were not reduced in treatment exposed mosquitoes at other time points (94.8% in treatment chambers, 92.6% in control), and model outputs indicate that treatment exposed mosquitoes blood fed at a reduced rate compared to controls (RR: 0.92 [0.87–0.98], p = 0.013) (S2 Table). Models estimate this effect diminishes after device aging (weekly “device age” RR: 1.02 [1.00–1.05]), and the raw data reveals no impact on blood feeding rates at one week after opening (97.1% feeding rate in mosquitoes from treatment chambers, 95.3% in control).

### Fecundity of blood fed female mosquitoes

Egg laying rates were higher among blood fed females from treatment chambers (median 8.4 eggs/mosquito, IQR: [6.1–14.7]) compared to individuals from control chambers (5.9 eggs/mosquito [4.8–10.2]) (Fig 6). Adult cage densities varied between groups (mean number of mosquitoes per control cage: 186; treatment: 125), with models indicating that the difference between egg-laying rates was significantly impacted by the adult cage density (RR: 0.61 [0.60–0.63], p < 0.001) and not overall treatment status (RR: 0.97 [0.92–1.02]). The egg hatch rate was similar between the eggs of control (median hatch rate: 0.52, IQR: [0.45–0.60]) and VPSR-exposed mosquitoes (median hatch rate: 0.54, IQR [0.47–0.67]) (RR: 1.39 [0.91–2.12], p = 0.13).

## Discussion

The endpoints collected in this trial were designed to measure the personal protection offered by a volatile pyrethroid-based spatial repellent (VPSR) containing transfluthrin, and other possible effects on *Anopheles gambiae* vectors that could provide community protection if applied at scale. In addition to landing rates measured by HLC, which have been significantly reduced by transfluthrin-based interventions in prior studies [24, 38, 39], an additional endpoint–deterrence–was measured by morning outdoor capture of living mosquitoes that were not captured throughout the night. These fates of non-host-seeking mosquitoes in the presence of the VPSR–and similar devices–are relevant in a field setting, with deterred mosquitoes possibly diverted to surrounding unprotected households in a manner that may be dose-dependent [26, 35]. The results from these semi-field trials indicate that the VPSR intervention is associated with a reduction of approximately 35–40% in overnight mosquito host-seeking behavior in chambers with freshly opened devices, with the effect declining slightly over time but persisting through the testing period lasting five weeks, with additional effects of increased deterrence and mortality compared to unexposed mosquitoes. Host-seeking reduction was observed at all time points, providing evidence for efficacy up to five weeks and suggesting possible efficacy beyond that period. Mosquito mortality was most strongly associated with fresh devices and mortality trended towards deterrence as the devices aged, possibly related to a dose response as the remaining transfluthrin in the devices declined. The intervention appeared to have little lasting impact on disarming blood feeding behavior, fecundity, or fertility. The device was tested in a configuration designed to protect local structures, but their transportability, compact design, and ease of use requiring no burning or power source are attributes which could make the VPSR appropriate for multiple use cases, including field-deployed military service members, recreation and tourism, outdoor events, and other instances when other forms of biting protection are not available. These results indicate that the intervention functions as expected through the primary mode of action in reducing landing, but the impacts are not limited to reduction in landing and the impact on disease transmission may be considered based on the accumulation of these effects.

It is possible that host-seeking behavior is over-estimated in this study, as the closed semi-field design forces non-host-seeking mosquitoes to remain within 10 meters of host-seeking cues from the human landing collectors throughout the night. In control chambers, hourly landing rates were higher in the first hour than all other hours, highlighting an hourly difference in host-seeking avidity that may be an artifact of the semi-field setting, considering that the natural circadian rhythms of *An*. *gambiae s*.*s*. generally peak after midnight. In models which consider the hourly HLC recapture rate, the predicted effect of the treatment is elevated roughly 50% above the observed all-night reduction of host-seeking, driven by the large difference in activity in the first hour which then “trails off” throughout the night (Fig 4). Maximum response in the first hour has been observed in other semi field studies [40, 41], and some authors have used multiple releases throughout the night to maintain mosquito biting pressure [42]. This straightforward change to semi-field design should be considered in future designs to investigate these hourly differences specifically to determine if they are more closely related to mosquito behavior within this closed system or product efficacy. With these considerations, the results of this study can be interpreted with the nightly efficacy acting as a more conservative estimate compared to the hourly results.

The secondary endpoints measured in this experiment were chosen to reflect outcomes of epidemiological importance in the field and from modeling studies [36]. Mosquito mortality or reductions in fitness have been observed in prior studies of transfluthrin [27–29], providing a mechanism for community protection through overall suppression of mosquito populations and reduced age structures [43, 44]. Deterrence and knockdown were both elevated in treatment chambers and may be dose-dependent, with the ratio of mortality to deterrence highest when testing fresh devices (Fig 5). Notably, the exposure-related mortality was largely observed in the perimeter ditches of treatment chambers. It is possible these mosquitoes were repelled by the devices and would have escaped in a natural setting but were prevented from doing so by the confines of the chamber, leading to over-estimation of mortality in this study. Their accumulation in the perimeter ditches also prevented the differentiation between mortality and knockdown effects. The location of deterred mosquitoes is noted in the results as slightly higher outside the structures in treatment huts. However, the study was not designed to differentiate between their location of capture, and all mosquitoes captured after HLC were considered deterred regardless of location as they did not host seek during the overnight period despite starvation.

The fates of host-seeking and deterred mosquitoes are also relevant in the context of community protection. In addition to the acute mortality/knockdown which may occur during exposure, delayed impacts on mosquito survival can contribute to community protection. In this study, mortality of host-seeking and deterred mosquitoes was observed for five days. A large increase in mortality was observed among “deterred” mosquitoes in both treated and untreated chambers after five days which was not present at one day, likely driven by the lack of a bloodmeal provided to these mosquitoes at the one-day time point. Mortality was significantly increased in VPSR-exposed HLC-captured mosquitoes at both time points, but this deleterious effect was very nearly significantly reversed among deterred mosquitoes after five days (OR: 0.34 [0.11–1.01], p = 0.053). This suggests that deterred mosquitoes could be less negatively impacted by the treatment compared to host-seeking mosquitoes, possibly due to lower exposure of active ingredient outside of the huts. It is also possible that–considering they missed twelve hours of feeding opportunities–the ‘deterred’ mosquitoes found in control chambers represent a particularly unfit subset of the original population, resulting in abnormally high mortality. This alternative explanation provides further support for a nightly multiple release experimental design. This deterrence effect should be further studied, as deterred mosquitoes appear capable of enduring intervention exposure and may divert to other nearby hosts. This finding also supports the ability of this semi-field system to estimate repellency and/or deterrence mechanisms, although an idealized design would be substantially larger than the expected area of effect of the tested device.

Prior studies of transfluthrin have utilized proxy measurements for blood feeding such as HLC [45], or allowed mosquitoes to freely bite to measure reductions in blood feeding [28]. It has been suggested in *Aedes* mosquitoes that landing and biting inhibition might differ [46], and separating these endpoints allows for host-seeking and probing behaviors to be considered separately. This disarming endpoint measured by a prolonged blood feeding inhibition even after exposure is particularly important to capture in semi-field systems, since biting behavior cannot be well quantified during field trials involving HLC or other trapping methods. In this study, blood feeding rates among host-seeking mosquitoes observed 12–24 hours post exposure were slightly, but significantly depressed in mosquitoes exposed to the VPSR devices, with the effect observed to entirely diminish by the first week after opening. This reduction in blood feeding behavior appears to be short-lived but should continue to be further studied in the presence or immediate aftermath of transfluthrin exposure, rather than 12–24 hours post exposure, to measure for how long after exposure mosquitoes are disarmed and if disarmament provides a community effect by delaying feeding cycles [27, 36]. Following successful feeding, egg-laying and hatch rates were slightly higher among treatment exposed mosquitoes overall but varied by device age without following a clear trend. Models suggested that both rates were driven by the mosquito density in experimental cages, which was considerably higher among control mosquitoes, rather than treatment status. The fate of hatched larvae was not followed, but additional generational effects such as fitness and sex ratio are endpoints that may be incorporated into an improved experimental design to understand these impacts.

Temperature and humidity also appear to play a role in mosquito behavior and VPSR efficacy, with higher nightly temperature and humidity associated with reduced efficacy in these experiments. In hourly analysis, higher humidity was associated with generally higher host-seeking across both chambers, while higher temperature and lowered humidity were associated with reduced efficacy. It is unclear why the association of humidity with efficacy is reversed in hourly and nightly analysis; it’s possible that it is a byproduct of improved model fit due to higher data resolution, or an indication of mosquito behavioral patterns. Overall, these experiments were conducted in cooler than optimal temperatures for volatile pyrethroids with a mean nightly temperature around 21C. Increased temperatures resulting in reduced transfluthrin efficacy contrasts with other studies that have shown improved efficacy at higher temperatures [26].

This study utilized a pyrethroid-susceptible strain of *An*. *gambiae s*.*s*., presenting multiple limitations and considerations for future semi-field studies. *An*. *gambiae s*.*s*. are generally highly endophilic and endophagic, while the semi-field system was deployed in an “outdoor” configuration with open-walled structures. The choice of species was largely a practical choice based on *Anopheles* species colony availability in Zambia and the scale of the trials necessitated the use of a stable and productive mosquito colony. Nonetheless, this species does not exhibit the behaviors observed in species which may be more relevant to outdoor transmission such as *An*. *arabiensis*. This is a limitation to the study which could be remedied with field trials in a natural setting including exophilic vectors, or further semi-field experiments using multiple species. Additionally, pyrethroid-susceptible mosquitoes were used in this study to provide a baseline estimate of effect, but it is difficult to make projections about the efficacy of this VPSR device without a better understanding of the impact of volatile transfluthrin in resistant mosquitoes. In previous lab, semi-field, and field studies, mosquitoes with metabolic resistance to pyrethroids have been inhibited by transfluthrin, but resistance to transfluthrin itself has not been studied [32–34].

## Conclusions

The results of this semi-field study suggest that the tested VPSR device, a passive emanator of the pyrethroid transfluthrin, demonstrates promise as a malaria control tool. Over the course of experimental nights, human landing was reduced at all time points over the five-week observation period after unsealing the devices, with evidence for heightened mortality transitioning towards deterrence effects over the use of the intervention. Landing rates were reduced up to and including five weeks past opening, with further duration of effect unknown from these trials. Overall rates of deterrence and mortality decreased over the five-week period in addition to the trend towards deterrence, suggesting a possible dose response. In general, there was very little impact on disarming, fecundity, or fertility, except in the case of fresh treatment devices where there was a small but significant decrease in blood feeding (a disarming effect). The data gathered in this study also support possible improvements for semi-field experimental design, primarily shorter recapture timeframes and repeated releases during experimental nights to better mimic a field environment where approaching mosquitoes are more consistently naïve to the active ingredient being tested. We also recommend that temperature and humidity is always monitored when evaluating the efficacy of personal protection tools.

## Supporting information

S1 Fig Nightly temperature and humidity mean values over the course of experiments in the semi-field system. Measurements were taken from a weather station (Onset HOBO) adjacent to the semi-field enclosure. Values are plotted on the same axis, with humidity reported as relative humidity (percentage) and temperature reported in Celsius.
(TIF)

S1 Table Model coefficients for primary experimental outcomes. All models were mixed effect generalized linear models (GLMER) with a Poisson (log) link function. Each model included the log-transformed number of mosquitoes released into the chamber as the exposure term, except for the hourly host-seeking model which uses the log-transformed number of mosquitoes remaining in the chamber at each hour. Models were assessed by AIC and coefficients which were dropped to enable model convergence are denoted with a dash ‘-‘. AIC and degrees of freedom for the null model are displayed in parenthesis after the values for each fitted model. Coefficients which are not relevant to a specific model are denoted with an NA. Date of experiment was included in all models as a random effect. P values are coded, with ‘***’ representing p values < 0.001, ‘**’ representing p values between 0.001 and 0.01, ‘*’ between 0.01 and 0.05, and ‘.’ representing nearly significant p values between 0.05 and 0.1. ^a^Temperature and Humidity were centered and scaled around their mean values for all models.
(DOCX)

S2 Table Model coefficients for delayed mortality and blood feeding behavior. All models were mixed effect generalized linear models (GLMER) with a binomial (logit) link function. Models were assessed by AIC and coefficients which were dropped to enable model convergence are denoted with a dash ‘-‘. AIC and degrees of freedom for the null model are displayed in parenthesis after the values for each fitted model. Coefficients which are not relevant to a specific model are denoted with an NA. Date of experiment was included in all models as a random effect. P values are coded, with ‘***’ representing p values < 0.001, ‘**’ representing p values between 0.001 and 0.01, ‘*’ between 0.01 and 0.05, and ‘.’ representing nearly significant p values between 0.05 and 0.1. ^a^Coefficients denoted with this symbol were centered and scaled around their mean values prior to model fitting. ^b^The age of treatment was considered as a numeric predictor in all models except the blood feeding model, where a binary factor (fresh vs not fresh) was used instead to better model the observed behavior.
(DOCX)

S1 Checklist Inclusivity in global research. (DOCX)

S1 File (DOCX)
