# Supplementary figures and images for: Semi-field evaluation of a volatile transfluthrin-based intervention reveals efficacy as a spatial repellent and evidence of other modes of action
Source: PLoS One. 2023 May 11;18(5):e0285501. doi: 10.1371/journal.pone.0285501 (PMC10174509; doi:10.1371/journal.pone.0285501)

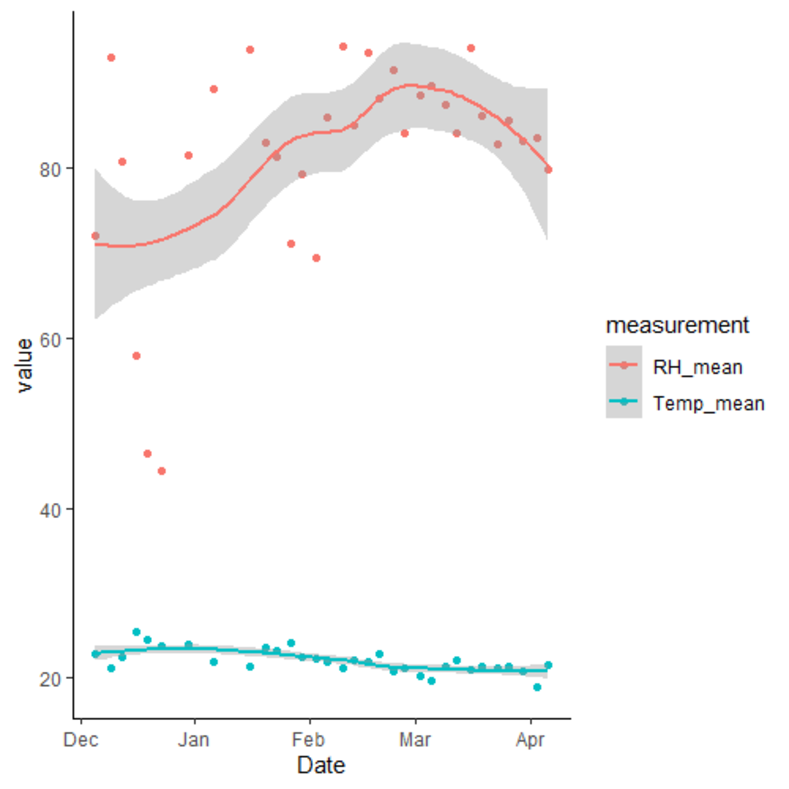

Supplement: S1 Fig — Measurements were taken from a weather station (Onset HOBO) adjacent to the semi-field enclosure. Values are plotted on the same axis, with humidity reported as relative humidity (percentage) and temperature reported in Celsius. (TIF) [file pone.0285501.s001.tif]
