# Supplementary material for: Semi-field evaluation of a volatile transfluthrin-based intervention reveals efficacy as a spatial repellent and evidence of other modes of action
Source: PLoS One. 2023 May 11;18(5):e0285501. doi: 10.1371/journal.pone.0285501 (PMC10174509; doi:10.1371/journal.pone.0285501)
Supplement: S1 Table — All models were mixed effect generalized linear models (GLMER) with a Poisson (log) link function. Each model included the log-transformed number of mosquitoes released into the chamber as the exposure term, except for the hourly host-seeking model which uses the log-transformed number of mosquitoes remaining in the chamber at each hour. Models were assessed by AIC and coefficients which were dropped to enable model convergence are denoted with a dash ‘-‘. AIC and degrees of freedom for the null model are displayed in parenthesis after the values for each fitted model. Coefficients which are not relevant to a specific model are denoted with an NA. Date of experiment was included in all models as a random effect. P values are coded, with ‘***’ representing p values < 0.001, ‘**’ representing p values between 0.001 and 0.01, ‘*’ between 0.01 and 0.05, and ‘.’ representing nearly significant p values between 0.05 and 0.1. aTemperature and Humidity were centered and scaled around their mean values for all models. (DOCX) [file pone.0285501.s002.docx]

**Table S1. Model coefficients for primary experimental outcomes.**

|  | DIRECT OUTCOMES – POISSON MODELS | | | | | | | | | |
| --- | --- | --- | --- | --- | --- | --- | --- | --- | --- | --- |
| Modeled endpoint | (A) Overall Recovery | | (B) Host-seeking (nightly) | | (C) HOST-SEEKING (hourly) | | (D) Deterrence | | (E) Mortality | |
| EXPOSURE TERM (“OFFSET”) | Num. released | | Num. released | | Num. remaining | | Num. released | | Num. released | |
| Fixed Effects |  |  |  |  |  |  |  |  |  |  |
| (Intercept) | **0.88 [0.84 – 0.93]** | ******* | **0.82 [0.76 – 0.88]** | ******* | **0.18 [0.15 – 0.20]** | ******* | **0.06 [0.05 – 0.07]** | ******* | **0.01 [0.01 – 0.02]** | ******* |
| Treatment (VPSR) | 0.95 [0.90 – 1.00] | **.** | **0.61 [0.57 – 0.65]** | ******* | **0.37 [0.34 – 0.40]** | ******* | **3.83 [3.30 – 4.44]** | ******* | **5.88 [4.50 – 7.68]** | ******* |
| Age of treatment | 1.00 [0.98 – 1.02] |  | 0.98 [0.96 – 1.01] |  | 0.96 [0.91 – 1.00] | **.** | 1.03 [0.92 – 1.16] |  | **1.24 [1.05 – 1.46]** | ***** |
| Chamber 2 | 1.02 [0.99 – 1.06] |  | 0.97 [0.94 – 1.01] |  | 1.03 [0.99 – 1.08] |  | **1.36 [1.23 – 1.49]** | ******* | 0.87 [0.73 – 1.04] |  |
| Temp. (nightly mEAN)^a^ | 0.97 [0.93 – 1.01] |  | 0.98 [0.92 – 1.04] |  | 0.96 [0.88 – 1.04] |  | **0.76 [0.66 – 0.88]** | *** | 1.11 [0.76 – 1.63] |  |
| RH % (nightly mean)^a^ | 1.03 [0.98 – 1.07] |  | 1.05 [0.99 – 1.11] |  | **1.11 [1.02 – 1.21]** | ***** | **0.85 [0.76 – 0.96]** | ****** | 1.01 [0.69 – 1.47] |  |
| Hour | NA |  | NA |  | **0.95 [0.95 – 0.96]** | ******* | NA |  | NA |  |
| Interactions |  |  |  |  |  |  |  |  |  |  |
| Treatment:Age | 1.00 [0.98 – 1.02] |  | **1.07 [1.05 – 1.10]** | ******* | **1.13 [1.10 – 1.16]** | ******* | **0.86 [0.81 – 0.91]** | ******* | **0.71 [0.65 – 0.78]** | ******* |
| TREATMENT:TEMP | 1.02 [0.97 – 1.07] |  | **1.10 [1.04 – 1.17]** | ******* | **1.08 [1.03 – 1.12]** | ******* | 1.01 [0.91 – 1.12] |  | 0.98 [0.79 – 1.23] |  |
| TREATMENT:RH | 0.99 [0.94 – 1.03] |  | **1.06 [1.00 – 1.12]** | ***** | **0.93 [0.89 – 0.97]** | ******* | - |  | 0.98 [0.80 – 1.22] |  |
| TREATMENT:HOUR | NA |  | NA |  | **1.02 [1.01 – 1.03]** | ****** | NA |  | NA |  |
|  |  |  |  |  |  |  |  |  |  |  |
| Degrees freedom | 50 (59) |  | 50 (59) |  | 708 (719) |  | 51 (59) |  | 50 (59) |  |
| AIC | 571 (607) |  | 594 (1033) |  | 7227 (9133) |  | 553 (1350) |  | 532 (1065) |  |
|  |  |  |  |  |  |  |  |  |  |  |

All models were mixed effect generalized linear models (GLMER) with a Poisson (log) link function. Each model included the log-transformed number of mosquitoes released into the chamber as the exposure term, except for the hourly host-seeking model which uses the log-transformed number of mosquitoes remaining in the chamber at each hour. Models were assessed by AIC and coefficients which were dropped to enable model convergence are denoted with a dash ‘-‘. AIC and degrees of freedom for the null model are displayed in parenthesis after the values for each fitted model. Coefficients which are not relevant to a specific model are denoted with an NA. Date of experiment was included in all models as a random effect. P values are coded, with ‘***’ representing p values < 0.001, ‘**’ representing p values between 0.001 and 0.01, ‘*’ between 0.01 and 0.05, and ‘.’ representing nearly significant p values between 0.05 and 0.1.

^a^Temperature and Humidity were centered and scaled around their mean values for all models.
