# Supplementary material for: Semi-field evaluation of a volatile transfluthrin-based intervention reveals efficacy as a spatial repellent and evidence of other modes of action
Source: PLoS One. 2023 May 11;18(5):e0285501. doi: 10.1371/journal.pone.0285501 (PMC10174509; doi:10.1371/journal.pone.0285501)
Supplement: S2 Table — All models were mixed effect generalized linear models (GLMER) with a binomial (logit) link function. Models were assessed by AIC and coefficients which were dropped to enable model convergence are denoted with a dash ‘-‘. AIC and degrees of freedom for the null model are displayed in parenthesis after the values for each fitted model. Coefficients which are not relevant to a specific model are denoted with an NA. Date of experiment was included in all models as a random effect. P values are coded, with ‘***’ representing p values < 0.001, ‘**’ representing p values between 0.001 and 0.01, ‘*’ between 0.01 and 0.05, and ‘.’ representing nearly significant p values between 0.05 and 0.1. aCoefficients denoted with this symbol were centered and scaled around their mean values prior to model fitting. bThe age of treatment was considered as a numeric predictor in all models except the blood feeding model, where a binary factor (fresh vs not fresh) was used instead to better model the observed behavior. (DOCX) [file pone.0285501.s003.docx]

**Table S2. Model coefficients for delayed mortality and blood feeding behavior.**

|  | Delayed outcomes – BINOMIAL MODELS | | | | | |
| --- | --- | --- | --- | --- | --- | --- |
| Modeled endpoint | **(A) 24 HOUR MORTALITY** | | **(A) FIVE DAY MORTALITY** | | **(C) POST EXPOSURE BLOODFEEDING** | |
| Fixed Effects |  |  |  |  |  |  |
| (Intercept) | **0.03 [0.01 – 0.04]** | ******* | **0.29 [0.22 – 0.38]** | ******* | **31.74 [18.27 – 55.14]** | ******* |
| Treatment (VPSR) | **2.42 [1.43 – 4.09]** | ******* | **1.50 [1.15 – 1.96]** | ****** | **0.50 [0.30 – 0.86]** | ***** |
| Age of treatment^b^ | 1.08 [0.97 – 1.19] |  | **-** |  | **0.39 [0.21 – 0.73]^%^** | ****** |
| Chamber 2 | **1.22 [1.04 – 1.43]** | ***** | - |  | **1.36 [1.15 – 1.62]** | ******* |
| Temp. (nightly mEAN)^a^ | **1.25 [1.01 – 1.55]** | ***** | **1.17 [1.02 – 1.33]** | ***** | **0.62 [0.43 – 0.89]** | ****** |
| RH % (nightly mean)^a^ | 1.04 [0.85 – 1.29] |  | 1.02 [0.89 – 1.16] |  | 0.76 [0.54 – 1.08] |  |
| Cage density^a^ | 1.26 [0.88 – 1.80] |  | 0.83 [0.69 – 1.01] | **.** | 1.02 [0.78 – 1.32] |  |
| Deterred  (vs HLC captured) | 3.25 [0.47 – 22.37] |  | **4.54 [1.58 – 13.02]** | ****** | NA |  |
|  |  |  |  |  |  |  |
| Interactions |  |  |  |  |  |  |
| Treatment:Age^b^ | 1.05 [0.96 – 1.14] |  | **-** |  | **3.90 [2.54 -5.99]^%^** | ******* |
| Treatment:deterred | 0.34 [0.05 – 2.51] |  | 0.34 [0.11 – 1.01] | **.** | NA |  |
| TREATmEnT:CAGE DEN.^a^ | **0.38 [0.23 – 0.64]** | ******* | **0.69 [0.52 – 0.90]** | ****** | **2.43 [1.78 – 3.32]** | ******* |
| deterred:Cage DEN.^a^ | 1.19 [0.18 – 7.98] |  | 1.00 [0.36 – 2.80] |  | 0.92 [0.82 – 1.04] |  |
| Treatment:Deterred:  Cage DEnsity^a^ | 2.40 [0.29 – 19.89] |  | 1.11 [0.35 – 3.50] |  | NA |  |
|  |  |  |  |  |  |  |
| Degrees freedom | 107 (119) |  | 109 (119) |  | 48 (57) |  |
| AIC | 629 (732) |  | 937 (2004) |  | 602 (1044) |  |

All models were mixed effect generalized linear models (GLMER) with a binomial (logit) link function. Models were assessed by AIC and coefficients which were dropped to enable model convergence are denoted with a dash ‘-‘. AIC and degrees of freedom for the null model are displayed in parenthesis after the values for each fitted model. Coefficients which are not relevant to a specific model are denoted with an NA. Date of experiment was included in all models as a random effect. P values are coded, with ‘***’ representing p values < 0.001, ‘**’ representing p values between 0.001 and 0.01, ‘*’ between 0.01 and 0.05, and ‘.’ representing nearly significant p values between 0.05 and 0.1.

^a^Coefficients were centered and scaled around their mean values prior to model fitting.

^b^The age of treatment was considered as a numeric predictor in all models except the blood feeding model, where a binary factor (fresh vs not fresh) was used instead to better model the observed behavior.
