# Supplementary material for: Semi-field evaluation of a volatile transfluthrin-based intervention reveals efficacy as a spatial repellent and evidence of other modes of action
Source: PLoS One. 2023 May 11;18(5):e0285501. doi: 10.1371/journal.pone.0285501 (PMC10174509; doi:10.1371/journal.pone.0285501)
Supplement: S1 File — (DOCX) [file pone.0285501.s005.docx]

**AIM:** to evaluate the efficacy of a spatial repellent (VPSR) deployed outdoors to repel mosquitoes.

Objectives: To measure protective efficacy again uninfected *An. gambiae* as

1. Reduced outdoor foraging (measured by human landing catches inside open walled kitchen structures)
2. Increased kill (measured by dead collections) or delayed kill (deaths of aspirated mosquitoes)
3. Secondary entomological endpoints: bloodfeeding inhibition, reduced oviposition, and reduced egg hatch rates

Variables to include in model: repellent presence, collection type, day (week), moon phase, climate, wind direction, chamber, collector.

**EQUIPMENT/SUPPLIES** needed for each night of experiments:

- 2 VPSR devices (2 sheets each, 4 total sheets)
- Papers and cups for egg-laying (4 per experiment)
- Larval pan (4 per experiment)

**EQUIPMENT/SUPPLIES** needed for experiment 1 (re-usable):

- 1x Plumb line (1.5m marked)
- 1x Labelling tape and marker pen
- Paper for labels
- Clip board with Collection form and counts form
- Pen
- Aspirators
- Larval food
- 28 paper cups – 2 per chamber for collecting live mosquitoes, and 24 for hourly HLC collections in each chamber – with netting rubber bands and cotton wool. Can be re-used (See SOP, rotate cups with VPSR device, as in always use “VPSR” cups to collect mosquitoes in the VPSR chamber, and “control” cups to collect mosquitoes in the control chamber.
- 6 Petri dishes – 3 per chamber for dead mosquitoes. Can be re-used.

**METHODS**

**EXPERIMENT 1**

**SET UP**

**Figure 1.1. Experiment setup for phase 1. Monday night layout shown, rotate new VPSR devices to chamber 2 for Thursday night layout. Red rectangles represent VPSR devices, hung from outside eaves of test hut as shown. HLC collections will occur within huts in chamber 1 and chamber 2.**


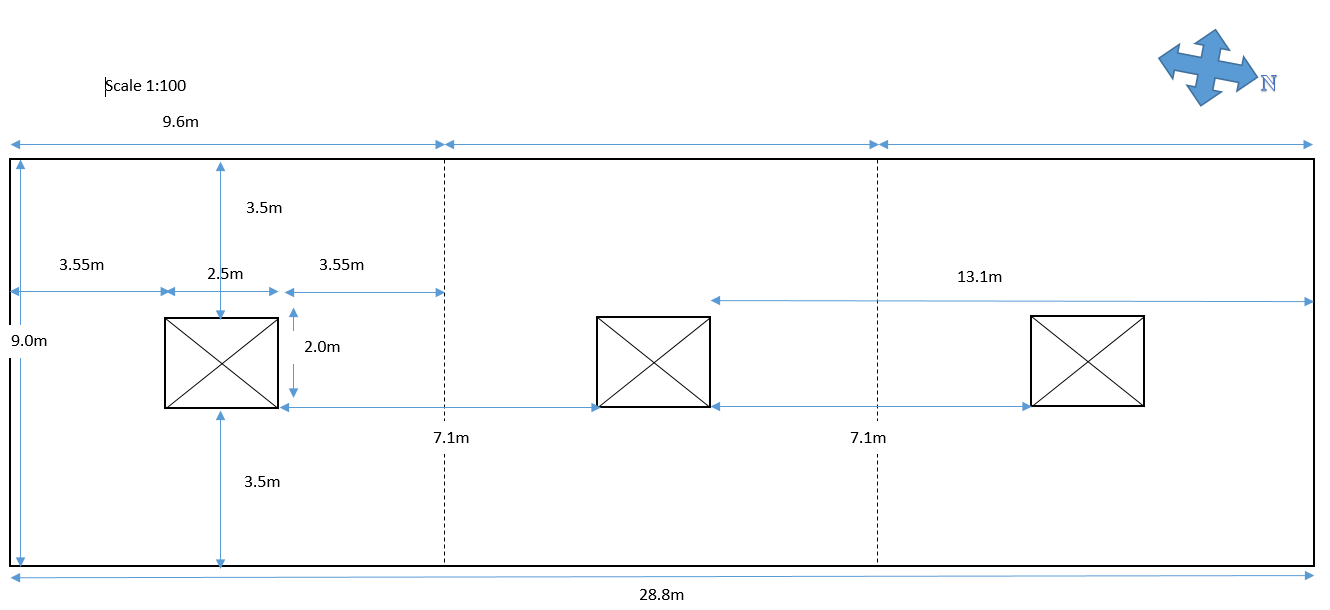


Chamber 3

Chamber 1

Experiment 1 will evaluate the effect of 2 deployed fresh VPSR devices compared to a control with nothing. Experiment will take place on the concrete side of the Mosquito House with the dividing walls rolled down. Middle chamber will be unused for this experiment, only chambers 1 and 3 will be used. Release of mosquitoes should take place two times per week on Monday and Thursday nights. VPSRS will be placed around the test hut on alternate collection nights e.g. Monday = VPSR in chamber 1, Thursday = VPSR in chamber 3.

PRIOR TO EXPERIMENT: PREPARATION OF COLLECTION CUPS

1. Label Petri dishes, 3 per chamber, with ‘VPSR Experiment 1 - dead’, VPSR or control, and location of collection– inside hut and outside hut, and in ditch (see counts form). Ex: **“VPSR Experiment 1 – dead, VPSR, inside hut”**
2. Label 24 paper cups with ‘VPSR Experiment 1 – HLC’, VPSR or control, and hour of collection (e.g. “1800-1900”). Seal with netting, rubber band, and cotton wool ready for HLC aspiration overnight. Ex: **“VPSR Experiment 1 – HLC, VPSR, 2000 – 2100”**
3. Label 4 paper cups with ‘VPSR Experiment 1 - live’, VPSR or control, and location of aspiration – inside hut and outside hut (see counts form). Seal with netting, rubber band and cotton wool ready for aspirations the next day. Ex: **“VPSR Experiment 1 - live, VPSR, inside hut”**

MOSQUITOES:

1. In the morning of the experiment collect 500 female non blood fed 2-5 day old *An. gambiae*  into 2 separate cages (2x 250 mosquitoes each). Provide glucose. **Do not proceed with testing unless there are at least 200 mosquitoes per cage.**
2. Record the experiment number, day (repetition of experiment), date of mosquito release, chamber number, presence of repellent or not, and the number of mosquitoes to be released in each chamber in **experiment form A, B.** For each night, each chamber should use a single row per form.

SET UP IN MOSQUITO HOUSE

1. Upper hut walls should be removed for these tests.
2. Ensure all chambers are ant and spider free. Sweep chamber out before and between experiments. Ensure trough is cleared of all mud, insects, spider webs and is filled with water half way with a bit of detergent. Ensure all netting is intact.
3. Lay down white sheeting in each chamber next to ant trough and in huts.
4. At 16:00, unwrap and place 2 new VPSR devices (**4 total VPSR sheets, one device is two sheets hung on one hanger)** on eaves of test hut on opposite sides of the hut (see diagram).
5. At 17:30, ensure a chair is in place for HLC collector in each chamber, and ensure cups with glucose for hourly collections (12 per chamber, labelled hourly), are ready and inside the mosquito sphere to be swapped by hour.
6. At 18:00 release one cage of 250 mosquitoes in chamber 1, and the other cage of 250 mosquitoes in chamber 2, ensuring none escape. Release mosquitoes along outside wall of each chamber (for chamber 1, along left wall upon entering, for chamber 3, upon right wall).

MOSQUITO COLLECTIONS

1. Each HLC collector should only ever do collections in one chamber (for example, collector A should always use chamber 1).
2. Enter each chamber and hut and begin HLC collections at 18:00.
3. Deposit aspirated HLC mosquitoes into appropriate cup (based on hour) as they land on the HLC collector. Cup should have glucose while mosquitoes are captured. Record mosquito numbers as they are captured.
4. Take a break at the end of each hour during collections for ~10 minutes, during this break move the collection cup for that hour into the insectary (make sure it has glucose). Use a new cup for each hour and provide glucose to cups with live mosquitoes.
5. Record HLC collection data into **Experiment form A** based on the hour of collection and chamber.
6. At 06:00 the HLC collectors in the huts should exit the chambers, ensuring that no mosquitoes escape.
7. At 0830 aspirate all live mosquitoes resting and flying. Per night have the 4 cups with glucose ready, 2 for chamber 1 and 2 for chamber 2. Aspirate those inside each hut into designated labelled cups, and aspirate those outside each hut into the designated cups. Record data as mosquitoes are captured and then copy to the appropriate rows and columns of **Experiment form B.** Keep mosquitoes to one side and provide glucose.
8. Collect all dead mosquitoes into 6 Petri dishes, one per location in chamber – from inside each hut, outside hut, and in water in ditch. Record data to appropriate rows and columns in **Experiment form B.**
9. Remove sheets and clean sphere.

CLEANING AND PREP OF CHAMBERS AND COLLECTION CUPS

1. Collect and deposit VPSRs in sealed plastic bag, clearly mark a large X on each bag of used VPSRs.
2. Store VPSRs until conclusion of all experiments, store separately from VPSRs that have not yet been used.
3. Once experiments are concluded, place all used VPSRs in a plastic bag, seal it, and incinerate (make sure they are completely burned).
4. After all collections have been made leave chambers for a day and a night to allow all mosquitoes if not collected to die. Sweep to remove dead insects.

PROCESSING OF COLLECTIONS

1. If not completed during HLC collection, add the numbers of mosquitoes captured during each hourly period and record the data in **Experiment form A** for both chambers.
2. After all collections are complete, combine mosquitoes captured by HLC into two cages, one cage for HLC mosquitoes from the VPSR chamber, and one cage each for HLC mosquitoes from the control chamber. Count number of mosquitoes in each cage and enter data in **Experiment form C, “# combined HLC”.** Place these two cages in the insectary and provide glucose. Keep emptied hourly collection cups for next round of experiment.
3. Combine mosquitoes from 4 cups of captured live mosquitoes into two cages, one cage for mosquitoes from the VPSR chamber, one cage for mosquitoes from the control chamber. Count number of mosquitoes in each cage and enter data in **Experiment form C, “# combined live capture”.** Place in insectary and provide glucose. Keep emptied collection cups for next round of experiment.
4. **Wash and air dry all cups and petri dishes used for HLC and capturing live/dead mosquitoes in the morning.** Keep labels on them and use them for the same cage next experiment night (for example VPSR cups should always be used in the chamber with VPSR, and never in the control chamber).

MONITORING BLOODFEED INHIBITION

1. During the day after the experiment night, provide unfed HLC and live collections mosquitoes from both VPSR and control chambers (4 total cages) with an anesthetized mouse for 30 minutes. Count # of bloodfed mosquitoes in each cage and enter data into **Experiment form C, “# HLC mosquitoes bloodfed” and “# Live capture mosquitoes bloodfed”**.

MONITORING DELAYED KILL

1. Monitor deaths from HLC collected mosquitoes from both chambers over a one-week period and document deaths in the HLC section of the ‘delayed kill’ counts form (**Experiment form D)**. As cages will build up over the time of the experiment ensure dates are well labelled on cups to be able to monitor deaths for each experimental day.
2. Monitor deaths from live-captured mosquitoes from both chambers over a one-week period and document deaths in the live-captured section of the ‘delayed kill’ counts form (**Experiment form D)**. As cages will build up over the time of the experiment ensure dates are well labelled on cups to be able to monitor deaths for each experimental day.

MONITORING OVIPOSITION AND HATCH INHIBITION

1. 3 days after the experiment night, provide bloodfed HLC and live collections mosquitoes from both VPSR and control chambers (4 total cages) with moistened filter paper in a petri dish for overnight oviposition. Record the date of egg laying in **Experiment form C.**
2. Count eggs the following morning from each cage and enter number into data form (**Experiment form C) “# Eggs laid (HLC)” and “# Eggs laid (live capture)”**.
3. Deposit eggs into fresh larval pans (1 larval pan for each cage, 4 total), provide pinch of larval food.
4. After two days, record date and count number of hatched larvae and enter number into data form **(Experiment form C), “# Larvae hatched (HLC)” and “# Larvae hatched (Live capture)”**.
5. **DO NOT PUT ANY HATCHED LARVAE FROM EXPERIMENT BACK INTO COLONY CAGES, KILL THEM.**
