# Supplementary material for: Semi-field evaluation of a volatile transfluthrin-based intervention reveals efficacy as a spatial repellent and evidence of other modes of action
Source: PLoS One. 2023 May 11;18(5):e0285501. doi: 10.1371/journal.pone.0285501 (PMC10174509; doi:10.1371/journal.pone.0285501)
Supplement: Attachment — Submitted filename: PONE-D-22-32222_reviewer.pdf [file pone.0285501.s006.pdf]

PLOS ONE

Semi-field evaluation of a volatile transfluthrin based intervention reveals efficacy as a spatial repellent and evidence of other modes of action

--Manuscript Draft--

**Manuscript Number:**	PONE-D-22-32222
**Article Type:**	Research Article
**Full Title:**	Semi-field evaluation of a volatile transfluthrin based intervention reveals efficacy as a spatial repellent and evidence of other modes of action
**Short Title:**	Semi-field evaluation of the Personal Insect Repellent Kit (PIRK), a volatile pyrethroid spatial repellent
**Corresponding Author:**	Timothy A Burton, B.S.<br>University of Notre Dame<br>Notre Dame, IN UNITED STATES
**Keywords:**	Entomology; repellents; intervention; malaria; Anopheles; pyrethroid; transfluthrin
**Abstract:**	Presently, malaria control tools are limited to targeting indoor biting and resting behaviors of Anopheles mosquito species. Few interventions are targeted towards malaria control in areas where transmission is driven or persists due to outdoor biting behaviors. The Personal Insect Repellent Kit (PIRK) is a passive volatile pyrethroid emanator designed to bridge this gap and provide protection from mosquito bites in outdoor spaces.<br>Southern Province, Zambia, is one such environment where outdoor biting is suspected to contribute to malaria transmission, where people are active in the evening in open-walled outdoor kitchens. This study assessed PIRK in replica kitchens within a controlled semi-field environment. Endpoints included effects on mosquito host seeking, immediate and delayed mortality, deterrence, blood feeding inhibition, and fertility.<br>Host-seeking was reduced by approximately 40% over the course of nightly releases in chambers containing PIRK devices. Mosquito behavior was not uniform throughout the night, and the modeled effect of PIRK was considerably higher when hourly catch rates were considered. These two observations highlight a limitation of this overnight semi-field design and consideration of mosquito circadian rhythms is recommended for future semi-field studies. Additionally, deterrence and immediate mortality were both observed in PIRK chambers, with evidence of delayed mortality and a dose related response. These results demonstrate a primarily personal protective mode of action with possible positive and negative community effects. Further investigation into this primary mode of action will be conducted through a field trial of the same product in nearby communities.
**Order of Authors:**	Timothy A Burton, B.S.
	Lewis Hambayi Kabinga
	Limonty Simubali
	Quinton Hayre
	Sarah J. Moore
	Jennifer C. Stevenson
	Neil F. Lobo
**Additional Information:**	
**Question**	**Response**
**Financial Disclosure**<br><br>Enter a financial disclosure statement that describes the sources of funding for the	This studywas sponsored by the Department of the Army, U.S. Army Contracting Command, Aberdeen Proving Ground, Edgewood Contracting Division, Ft Detrick MD[under Deployed Warfighter Protection (DWFP) Program Grant #W911QY1810001].

Powered by Editorial Manager® and ProduXion Manager® from Aries Systems Corporation

work included in this submission. Review the submission guidelines for detailed requirements. View published research articles from *PLOS ONE* for specific examples.<br><br>This statement is required for submission and **will appear in the published article** if the submission is accepted. Please make sure it is accurate.<br><br>**Unfunded studies**<br>Enter: *The author(s) received no specific funding for this work.*<br><br>**Funded studies**<br>Enter a statement with the following details:<br>• Initials of the authors who received each award<br>• Grant numbers awarded to each author<br>• The full name of each funder<br>• URL of each funder website<br>• Did the sponsors or funders play any role in the study design, data collection and analysis, decision to publish, or preparation of the manuscript?<br>• **NO** - Include this sentence at the end of your statement: *The funders had no role in study design, data collection and analysis, decision to publish, or preparation of the manuscript.*<br>• **YES** - Specify the role(s) played.<br><br>* typeset	
**Competing Interests**<br><br>Use the instructions below to enter a competing interest statement for this submission. On behalf of all authors, disclose any competing interests that could be perceived to bias this work—acknowledging all financial support and any other relevant financial or non-financial competing interests.<br><br>This statement is required for submission and **will appear in the published article** if the submission is accepted. Please make sure it is accurate and that any funding sources listed in your Funding Information	The authors have declared that no competing interests exist.

later in the submission form are also declared in your Financial Disclosure statement.<br><br>View published research articles from *PLOS ONE* for specific examples.<br><br>**NO authors have competing interests**<br><br>Enter: *The authors have declared that no competing interests exist.*<br><br>**Authors with competing interests**<br><br>Enter competing interest details beginning with this statement:<br><br>*I have read the journal's policy and the authors of this manuscript have the following competing interests: [insert competing interests here]*<br><br>* typeset	
**Ethics Statement**<br><br>Enter an ethics statement for this submission. This statement is required if the study involved:<br><br>• Human participants<br>• Human specimens or tissue<br>• Vertebrate animals or cephalopods<br>• Vertebrate embryos or tissues<br>• Field research<br><br>Write "N/A" if the submission does not require an ethics statement.<br><br>General guidance is provided below. Consult the submission guidelines for detailed instructions. **Make sure that all information entered here is included in the Methods section of the manuscript.**	This study was approved by the institutional review board at the University of Notre Dame (Protocol #: 18-05-4675) and by the local IRB at Macha Research Trust (IRB #: IRB0007649). Study participants were Macha Research Trust entomology staff who were fully informed of the risks and voluntarily provided informed oral consent. Their employment was not contingent on participation in the study. They did not receive additional compensation or incentives for the study but were paid at their normal pay rates for their work.

Powered by Editorial Manager® and ProduXion Manager® from Aries Systems Corporation

**Format for specific study types**<br><br>**Human Subject Research (involving human participants and/or tissue)**<br>• Give the name of the institutional review board or ethics committee that approved the study<br>• Include the approval number and/or a statement indicating approval of this research<br>• Indicate the form of consent obtained (written/oral) or the reason that consent was not obtained (e.g. the data were analyzed anonymously)<br><br>**Animal Research (involving vertebrate animals, embryos or tissues)**<br>• Provide the name of the Institutional Animal Care and Use Committee (IACUC) or other relevant ethics board that reviewed the study protocol, and indicate whether they approved this research or granted a formal waiver of ethical approval<br>• Include an approval number if one was obtained<br>• If the study involved *non-human primates*, add *additional details* about animal welfare and steps taken to ameliorate suffering<br>• If anesthesia, euthanasia, or any kind of animal sacrifice is part of the study, include briefly which substances and/or methods were applied<br><br>**Field Research**<br><br>Include the following details if this study involves the collection of plant, animal, or other materials from a natural setting:<br>• Field permit number<br>• Name of the institution or relevant body that granted permission	
**Data Availability**<br><br>Authors are required to make all data underlying the findings described fully available, without restriction, and from the time of publication. PLOS allows rare exceptions to address legal and ethical concerns. See the PLOS Data Policy and FAQ for detailed information.	Yes - all data are fully available without restriction

A Data Availability Statement describing where the data can be found is required at submission. Your answers to this question constitute the Data Availability Statement and **will be published in the article**, if accepted.<br><br>**Important:** Stating 'data available on request from the author' is not sufficient. If your data are only available upon request, select 'No' for the first question and explain your exceptional situation in the text box.<br><br>Do the authors confirm that all data underlying the findings described in their manuscript are fully available without restriction?	
**Describe where the data may be found in full sentences. If you are copying our sample text, replace any instances of XXX with the appropriate details.**<br><br>• If the data are **held or will be held in a public repository**, include URLs, accession numbers or DOIs. If this information will only be available after acceptance, indicate this by ticking the box below. For example: *All XXX files are available from the XXX database (accession number(s) XXX, XXX.)*.<br>• If the data are all contained **within the manuscript and/or Supporting Information files**, enter the following: *All relevant data are within the manuscript and its Supporting Information files.*<br>• If neither of these applies but you are able to provide **details of access elsewhere**, with or without limitations, please do so. For example:<br><br>*Data cannot be shared publicly because of [XXX]. Data are available from the XXX Institutional Data Access / Ethics Committee (contact via XXX) for researchers who meet the criteria for access to confidential data.*<br><br>*The data underlying the results presented in the study are available from (include the name of the third party*	All relevant data are within the manuscript and its Supporting Information files.

and contact information or URL).<br>• This text is appropriate if the data are owned by a third party and authors do not have permission to share the data.<br><br>* typeset	
Additional data availability information:	

Powered by Editorial Manager® and ProduXion Manager® from Aries Systems Corporation

Manuscript Click here to access/download;Manuscript;Manuscript.docx

Semi-field evaluation of a volatile transfluthrin based intervention reveals efficacy as a spatial repellent and evidence of other modes of action

Timothy A. Burton[1*], Lewis Hambayi Kabinga[2], Limonty Simubali[2], Quinton Hayre[1], Sarah J. Moore[3,4,5,6], Jennifer C. Stevenson[2,7], Neil F. Lobo[1]

[1] Eck Institute for Global Health, University of Notre Dame, Notre Dame, IN, USA.

[2] Macha Research Trust, Choma District, Zambia.

[3] Vector Control Product Testing Unit (VCPTU), Ifakara Health Institute, Environmental Health, and Ecological Sciences

[4] Vector Biology Unit, Department of Epidemiology and Public Health, Swiss Tropical & Public Health Institute

[5] Faculty of Science, University of Basel, Petersplatz 1, 4001 Basel, Switzerland;

[6] The Nelson Mandela African Institution of Science and Technology (NM-AIST), P.O. Box 447, Tengeru, Arusha, Tanzania

[7] Department of Molecular Microbiology and Immunology, Bloomberg School of Public Health, Johns Hopkins University, Baltimore, MD, USA.

* Corresponding author

E-mail: tburton@nd.edu (TAB)

Author contributions:

TAB: Data Curation, Formal Analysis, Investigation, Methodology, Visualization, Project Administration, Writing – Original Draft Preparation, Review & Editing. LHK: Investigation, Project Administration, Resources. LS: Investigation, Project Administration, Resources. QH: Data Curation, Formal Analysis. SJM: Methodology, Writing – Review & Editing. JCS: Conceptualization, Methodology, Supervision, Writing – Review & Editing. NFL: Conceptualization, Funding Acquisition, Methodology, Supervision, Writing – Review & Editing

Abstract

Presently, malaria control tools are limited to targeting indoor biting and resting behaviors of *Anopheles* mosquito species. Few interventions are targeted towards malaria control in areas where transmission is driven or persists due to outdoor biting behaviors. The Personal Insect Repellent Kit (PIRK) is a passive volatile pyrethroid emanator designed to bridge this gap and provide protection from mosquito bites in outdoor spaces.

Southern Province, Zambia, is one such environment where outdoor biting is suspected to contribute to malaria transmission, where people are active in the evening in open-walled outdoor kitchens. This study assessed PIRK in replica kitchens within a controlled semi-field environment. Endpoints included effects on mosquito host seeking, immediate and delayed mortality, deterrence, blood feeding inhibition, and fertility.

Host-seeking was reduced by approximately 40% over the course of nightly releases in chambers containing PIRK devices. Mosquito behavior was not uniform throughout the night, and the modeled effect of PIRK was considerably higher when hourly catch rates were considered. These two observations highlight a limitation of this overnight semi-field design and consideration of mosquito circadian rhythms is recommended for future semi-field studies. Additionally, deterrence and immediate mortality were both observed in PIRK chambers, with evidence of delayed mortality and a dose related response. These results demonstrate a primarily personal protective mode of action with possible positive and negative community effects. Further investigation into this primary mode of action will be conducted through a field trial of the same product in nearby communities.

Introduction

Vector control measures have a large impact on malaria burden, accounting for an estimated 81% of total malaria reduction between 2000-2015 (1). The existing interventions, long lasting insecticide-treated nets (LLINs) and indoor residual spraying (IRS), counter indoor late-night biting and indoor resting behaviors. These bionomic traits are generally common in many key *Anopheles* vectors of malaria including *An. gambiae s.s.* and *An. funestus,* the primary vectors in much of sub-Saharan Africa. However, susceptible biting patterns have been observed to shift in response to interventions (2–4). In addition, molecular identification has revealed unexpectedly high *Anopheles* species diversity in

many settings demonstrating greater complexity in transmission dynamics (5–7). These shifts in species compositions, densities, and bionomic traits are not well documented and may be highly spatially heterogenous, with one model estimating that Africans in general are experiencing 10% fewer of their mosquito bites while in bed or indoors in 2018 compared to 2003 (8). In many settings, this shift in biting patterns has resulted in increased mosquito exposure outdoors or earlier in the evening (4,9–14). For vector control efforts, these behavioral shifts and other gaps in protection are identifiable in many settings and are becoming more relevant to malaria transmission in areas where much of the malaria burden has been reduced such as Zambia's Southern Province where this study was conducted.

Community protection is a vital aspect of existing interventions including LLINs and IRS; by reducing local mosquito populations and therefore exposure of the community as a whole, the insecticidal action of these interventions can provide significant protection to a user's unprotected neighbors (15). This community effect somewhat depends on the resistance status of local mosquitoes, and is a vital aspect of the continued effectiveness of these interventions (16–19). While LLINs and IRS provide significant community effects through control of local malaria vectors, they both specifically target indoor biting and resting behaviors. Additional tools that target other mosquito behaviors may be required to provide personal and community protection in additional settings. There are no official recommendations for interventions to be deployed in these spaces, such as outdoors and in the early evening, where people may be at risk of mosquito biting (20). Structural improvements, such as closing eaves and screening windows and doorways of homes, are possible alternatives to traditional indoor interventions but are impractical or inapplicable for many peridomestic and outdoor spaces (21). Larval source management allows public health officials to reduce local mosquito populations, but is impractical over large areas and doe[illegible]t directly target outdoor biting (20,22).

Outdoor transmission is relevant in many low malaria settings, due in part to human behaviors in the early evening and morning which can expose them to mosquito activity (4,23). Outdoor human-mosquito interactions are quite variable, highlighting the advantages of flexibility, portability, and ease of use in interventions that target these behaviors. Volatile pyrethroid-based spatial repellent (VPSR) interventions incorporate these advantages and have shown promise in reducing mosquito landing behavior in prior studies (24–26), in addition to increasing mosquito mortality (27–29), and some evidence of reducing blood feeding behavior (27). The Personal Insect Repellent Kit (PIRK) is a volatile pyrethroid spatial repellent (VPSR) developed by Widder Bros., Inc. The pyrethroid active ingredient

transfluthrin has been shown to cause mortality/knockdown effects in addition to repellency in *Anopheles* mosquitoes (25,30,31). These outcomes are of epidemiological importance, with the undesirable possibility of repelled mosquitoes diverting to nearby hosts (32), and knockdown or kill effects possibly reducing transmission in a mechanism similar to the community effect provided by LLINs and IRS (33).

This study used a semi-field system at Macha Research Trust, southern Zambia, to evaluate the PIRK with entomological outcomes in mind. The system is in a setting with seasonal and very low malaria transmission, with outdoor mosquito biting behavior and high ITN coverage. Much of the outdoor biting is thought to occur in the evenings and mornings while families are gathered in open-walled kitchen huts. The PIRK devices could therefore reduce mosquito interactions in these spaces. The study design utilized the semi-field enclosure to conduct controlled release and recapture experiments to measure endpoints beyond mosquito landing. Study endpoints were designed to measure landing rate, repellency, and knock down during active PIRK use, in addition to delayed effects on blood feeding, mortality, and fecundity after exposure. These study endpoints were employed to assess the efficacy and longevity of PIRK devices.

Materials and Methods

Ethical statement

This study was approved by the institutional review board at the University of Notre Dame (Protocol #: 18-05-4675) and by the local IRB at Macha Research Trust (IRB #: IRB0007649). Study participants were Macha Research Trust entomology staff who were fully informed of the risks and voluntarily provided informed oral consent. Their employment was not contingent on participation in the study. They did not receive additional compensation or incentives for the study but were paid at their normal pay rates for their work.

Intervention

The Personal Insect Repellent Kit (PIRK) is a volatile pyrethroid spatial repellent (VPSR) developed by Widder Bros., Inc. (Fig 1). Each device consists of two 25x25cm sheets and serves as a passive emanator of the volatile

pyrethroid transfluthrin to provide an area of protection from mosquito activity for up to a month or longer without replacement. The devices were deployed within the semi-field kitchen structures by hanging them under two opposite eaves of the structure, 1.5 meters from the floor (Fig 1). Two devices were deployed to each kitchen (one per side).

Semi-field system

The semi-field system is a large enclosure walled with a fine mesh to prevent the ingress of local insects or the egress of test mosquitoes whilst allowing temperatures to largely equilibrate with the external environment (Fig 1). The two test chambers are separated by an unused third test chamber, and each chamber is isolated from the other by interior walls made of the same fine mesh material. Each chamber measures approximately 10m by 10m, with a lower ceiling of fine mesh about 3m above the ground. The entire enclosure sits under a simple sealed plastic roof for protection from the elements. Each chamber is surrounded by a narrow ditch filled with water and a mild surfactant, which prevents crawling insects that might prey on mosquitoes from reaching the interior of the chambers. During the experiments, the cement floors of the chambers were covered in white cloth which was wetted with water before each experiment. These cloths served to increase the relative humidity within chambers while providing a backdrop to easily find dead mosquitoes. This design allowed for the simultaneous evaluation of PIRK against a baseline control using mosquitoes reared from the same generation in an insectary.

Fig 1. Structures and PIRK deployment. Floorplan of semi-field chambers used for nightly release-recapture experiments (A). The middle chamber was not used for this study. PIRK devices deployed in a semi-field replica structure with the hut in an outdoor configuration (upper walls removed). Example of a typical outdoor kitchen in the area (C).

The test huts located in each chamber represent shelters used in the area. For this study, the upper half of test shelter walls were removed to replicate the design of local kitchen shelters (Fig 1). These shelters have 2m x 2.5m floors, with brick walls roughly 1m tall except in the doorway, an additional 1m of open sides, and a grass roof.

Study design

This experiment used a simple 2x2 Latin square rotational design between the test chamber (PIRK) and control chamber (negative control) to account for chamber and weekday effects. The human collectors stayed in the same

chamber on each night to enable the collector and chamber effects to be coupled as a single source of bias. Experiments were conducted every third or fourth night (on Monday and Thursday nights) to provide a wash out period between replicates and allow the transfluthrin and any host associated odors to dissipate between rotations.

Experiments were conducted from December 2019 through April 2020. Temperature and humidity were recorded at 5-minute intervals for the duration of experimental nights using a data logger (Onset HOBO). External rainfall and moon phase were recorded categorically for each night. Experiments took place over 32 nights, including two nights of baseline collection with no PIRKs in place. Ten nights were dedicated to testing ten separate fresh sets of PIRK devices. Four of these sets were tested weekly for five weeks over the remaining twenty nights. Between timepoints, these devices were kept freely hanging out of direct sunlight above an open office window.

Mosquitoes and insectary conditions

Colony-reared *Anopheles gambiae s.s.* (Kisumu strain) were used for all experiments. This colony is well established at MRT and is susceptible to all pyrethroid insecticides (unpublished data). Mosquitoes are reared in large cages and experimental groups were kept in 30cm x 30cm BugDorm cages. Reared mosquitoes were provided with 10% sucrose solution ad libitum. These cages were held in the insectary at 27 degrees C and 80% humidity until transfer to the experimental chambers. Approximately 250-300 2–5-day old female mosquitoes were selected from these colonies for release into per experimental chamber on a given night. Sucrose was removed from cages to starve experimental mosquitoes four hours prior to release.

Cage setup

Mosquitoes were selected in the early afternoon and placed into separate cages for each chamber before experiments commenced. PIRK devices were deployed to the appropriate chamber before 17:00, while the opposite chamber served as a no-device control. At 17:00, the chambers were prepared for the nightly replicate. This included filling the perimeter troughs with water and a mild surfactant and laying out and wetting white cloth on the floors of each chamber. At 18:00, mosquitoes were moved to the experimental chambers from the insectary and released from their cages, signaling the start of an experimental replicate (Fig 2).

Fig 2. Timeline of experimental replicates. Experimental endpoints are indicated in gray boxes. Bloodmeals were offered to host-seeking mosquitoes at 1-day post-exposure, 24 hours after mosquitoes were released into experimental chambers. Egg-laying rates were measured overnight after 4-days post-exposure, with hatch rates measured the following morning. Delayed mortality was noted for all days starting at 1-day post-exposure for deterred and host-seeking mosquitoes.

Endpoints

Study Endpoints

Endpoint	Definition	Model output
**Landing/Host-seeking**	Mosquito exhibits host-seeking behavior and lands on the mosquito collector	Risk Ratio
**Disarming**	Blood feeding rate of host-seeking mosquitoes	Odds Ratio
**Fecundity**	Egg laying and hatch rates of fed mosquitoes	Risk Ratio
**Delayed Mortality (Landing population)**	Landing mosquito death up to five days after exposure	Odds Ratio
**Deterrence**	Mosquito does not exhibit host-seeking behavior overnight, recovered alive in the morning	Risk Ratio
**Delayed Mortality (Deterred population)**	Deterred mosquito death up to five days after exposure	Odds Ratio
**Knockdown/mortality**	Mosquito does not exhibit host-seeking behavior overnight, recovered dead in the morning	Risk Ratio

Table 1. Definitions of study endpoints. The model output of each endpoint is noted based on the model family used.

Host-seeking behavior

Mosquitoes were recaptured from within the shelters by trained entomologists performing human landing collection (HLC). This is the gold standard for mosquito collection, where collectors use a mouth aspirator to collect mosquitoes that land on them (34). Collections took place overnight, with mosquitoes collected and counted separately by hour from 18:00-06:00. Collectors were provided coffee and listened to music or radio to aid in staying awake. All mosquitoes caught by HLC were moved to the insectary at the end of each hour for additional experiments. Host seeking behavior measured by HLC constituted the primary experimental endpoint and was calculated per night by dividing the

total HLC recapture in a chamber by the number of mosquitoes released. Hourly host seeking behavior was calculated by dividing the HLC recapture for a single hour by the number of mosquitoes remaining in the chamber at the start of that hour.

Deterrence and Mosquito Knockdown/Mortality

Following HLC collections, additional collectors entered each chamber at 08:00 and collected all remaining alive and dead mosquitoes. Two collectors per chamber actively searched for remaining mosquitoes using mouth aspirators, with one searching inside the hut and the other the remaining portions of the chamber including the perimeter ditch. Collectors rotated between chambers halfway through at 09:00, finishing clearing chambers at 10:00. The locations of these mosquitoes were noted as inside/outside the shelter, with survivors moved to the insectary and knocked down mosquitoes counted and discarded. Deterrence was informed by the proportion of released mosquitoes found alive outside of the hut in these morning collections. Knocked down or dead mosquitoes were not monitored for recovery, and direct mosquito mortality was calculated as the proportion of released mosquitoes found knocked down or dead.

Overall recovery rate

The overall recovery rate from chambers was included as a general measure of experimental bias and to detect effects which were not captured by study endpoints. The overall recovery rate was calculated by dividing the sum of mosquitoes recovered from the chamber (host-seeking, deterred, and knocked down) by the number of mosquitoes released into the chamber the previous night. On a small number of experimental nights (n = 5/64), this recovery rate was slightly higher than 100%, possibly due to miscounting or mosquito survival inside the chambers between replicates. For these chambers, the number of released mosquitoes was amended to yield a recovery rate of 100%.

Delayed effects

In the insectary, HLC-captured and deterred mosquitoes were separately followed for five days post-exposure (d.p.e.) to measure delayed mortality effects of PIRK exposure. Additionally, host seeking (HLC captured) mosquitoes were offered a bloodmeal from an anesthetized mouse at roughly 18:00, or 12-24-hours post-capture to measure inhibition of blood feeding behavior (disarming). These mosquitoes were sugar-starved for four hours prior to the

bloodmeal. The numbers of bloodfed females were counted and provided wetted filter papers for egg laying three days later (Fig 2). The numbers of laid eggs were counted and deposited into fresh larval pans; the hatched larvae were counted the next day and discarded. These data were used to post-exposure blood feeding, egg laying (fecundity), and egg hatch rates (fertility).

Statistical analysis

Generalized linear mixed models with an appropriate error distribution and a log link function were used for analysis. Host seeking, knockdown, and deterrence were analyzed using a Poisson distribution, which included an offset term to adjust for the number of mosquitoes exposed to the outcome (e.g., released into each chamber). Fixed effects included treatment and the age (in weeks after opening) of the PIRK device, chamber, temperature, and humidity with the date of experiment included as a random effect to account for day-to-day variation in mosquito behavior and other unmeasured factors contributing to experimental variation. An interaction between treatment and PIRK age was added to measure the effect of time on PIRK efficacy. Model coefficients were exponentiated and reported as rate ratios with the control set as the reference. The remaining endpoints gathered in the insectary (blood feeding, delayed mortality, fecundity) were modeled using a binomial distribution with the addition of cage density as a fixed effect. Models were evaluated and selected based on Akaike information criterion (AIC), with some fixed effects dropped from specific models when their addition reduced model fit. All data analysis was conducted in R version 4.0.2. Data was cleaned, summarized, and plotted using the tidyverse packages ‘tidyr’, ‘dplyr’, and ‘ggplot2’. Generalized linear models were generated and analyzed with the ‘lme4’ and ‘arm’ packages.

Results

Over the course of data collection from December 2019 through April 2020, the mean temperature during the hours of experiments was 21 degrees C (s.d. = 1.6), decreasing slightly over time. Mean nightly humidity was 83.9% (s.d. = 13.5%), only dipping below 75% for a few nights in December and early 2020. In general, the early period had warmer and sometimes dryer nights (Fig S1).

Recovery of released mosquitoes and sources of bias

A total of 8885 mosquitoes were released across 34 nights (261/night) in untreated chambers, compared to 7848 across 30 nights (262/night) in treatment chambers. The additional four nights represent a baseline period which was not included in comparison models. An identical number of mosquitoes were released into both chambers on a given night. The overall recovery rate, defined as the number of mosquitoes that were recovered from the chambers by all experimental endpoints, was similar between treatment (84.9%) and control (89.7%) (RR: 0.95 [0.90 – 1.00], $p = 0.061$), as well as between chambers (86.4% vs 88.5%; (RR: 1.02 [0.99 – 1.06], $p = 0.25$). There was no observed significant effect of treatment, chamber/volunteer, or any other predictors on the recovery rate (full model in Table S1).

Host-seeking behavior of mosquitoes – protective efficacy

In total, 7033/7974 (88.2%) of mosquitoes released in control chambers were captured by HLC, compared to 4319/6662 (64.8%) in PIRK chambers. The greatest protective efficacy (PE) was observed using fresh PIRK devices, with PE remaining but generally decreasing through the five tested weeks (Fig 3 and Fig 4). PIRK was associated with reduced mosquito landings across all timepoints when analyzed by night (RR: 0.61 [0.57 – 0.65], $p < 0.001$), or by hour (RR: 0.37 [0.34 – 0.40], $p < 0.001$). Overall, mosquito host-seeking across all chambers decreased later in the night (hourly RR: 0.95 [0.95 – 0.96], $p < 0.001$). The reduction of host seeking associated with PIRK declined slightly as experiments progressed into the night (hourly RR: 1.02 [1.01 – 1.03], $p = 0.003$). Both analyses showed a decline in PIRK efficacy in the weeks after opening (weekly "PIRK age" RR: 1.07 [1.05 – 1.10], $p < 0.001$ based on the all-night model; RR: 1.13 [1.10 – 1.16], $p < 0.001$ based on the hourly model).

Fig 3. Protective efficacy during overnight human landing in PIRK chambers relative to control chambers. The Y axis displays the risk ratio associated with PIRK exposure, calculated by dividing the proportion of mosquitoes captured through the night in PIRK chambers by the corresponding proportion in control chambers. The calculated RR has been subtracted from 1 to display protective efficacy as increasing along the Y-axis. Results are separated by age category of the PIRK device on the X axis. The dotted horizontal line refers to a ratio of 1 (no change between groups) with increasing values on the Y axis referring to increased protective efficacy.

Fig 4. Cumulative overnight proportion of recapture by human landing collection in semi-field experiments of released mosquitoes in PIRK and treatment chambers. The proportion of mosquitoes recaptured in treatment and control

chambers is displayed on the Y axis cumulatively by hour along the X axis. Each panel represents experimental nights with the corresponding PIRK age in weeks, up to five weeks past opening.

Nightly temperature and humidity had no effect on control HLC recapture rates, but both were associated with reduced overnight PIRK efficacy (scaled temperature RR: 1.10 [1.04 – 1.17], $p < 0.001$; scaled humidity RR: 1.06 [1.00 – 1.12], $p = 0.04$). This is likely because higher scaled hourly humidity was associated with greater overall mosquito activity (RR: 1.11 [1.02 – 1.21], $p = 0.01$). Higher hourly temperature was associated with reduced hourly PIRK efficacy (scaled temperature RR: 1.08 [1.03 – 1.12], $p < 0.001$), while higher hourly humidity was associated with increased efficacy (scaled humidity RR: 0.93 [0.89 – 0.97], $p < 0.001$). Chamber had no overall effect on human landing rate. Full model coefficients are provided in Table S1.

Deterrence

In total, 8.5% (n = 680/7974) of mosquitoes in control chambers were found alive the next morning. Of these, 60% (n = 411/680) were found outside the huts. Comparatively, 25.8% (n = 1717/6662) of mosquitoes were alive in test chambers, and 77% (n = 1321/1717) of these were found outdoors. The presence of PIRK substantially increased deterrence (RR: 3.83 [3.30 – 4.44], $p < 0.001$), and the effect declined with weekly PIRK age (RR: 0.86 [0.81 – 0.91], $p < 0.001$) (Table S1).

Knockdown

Mosquito knock-down was also elevated in treatment chambers (9.4% of all recovered mosquitoes, n = 626/6662)) compared to control chambers (3.3%, n=261/7974). The largest difference was observed using freshly opened PIRKs (15.5% in test chambers vs 2.3% in control), and these effects subsided in the five weeks after opening. Overall, there was no observable chamber effect on knock-down (6.1% vs 6.0% between chambers for all experimental nights). PIRK was associated with a substantial increase in the proportion of mosquitoes knocked down (RR: 5.88 [4.50 – 7.68], $p < 0.001$), with the effect decreasing with PIRK age (weekly “PIRK age” RR: 0.71 [0.65 – 0.78], $p < 0.001$) (Table S1). The ratio of deterrence relative to knockdown increased with weekly PIRK age, with fresh PIRK devices resulting in

higher knockdown relative to deterrence compared to older devices, which trend towards heightened deterrence overall (Fig 5).

Fig 5. Ratio of deterrence compared to mortality associated with PIRK exposure in the semi-field system. The ratio of deterrence (captured alive outdoors and abbreviated to "Det.") between PIRK and control chambers was divided by the ratio of knockdown (KD) between PIRK and control chambers and plotted on the y axis. Results were separated by age category on the x axis. The dotted horizontal line refers to a ratio of 1 (no change between groups). Ratios greater than one indicate higher deterrence relative to mortality.

Post-exposure survival of host-seeking and deterred mosquitoes

The survival of mosquitoes recaptured while host-seeking (HLC captured) and moving away from the PIRK (deterred) was observed separately for five days post-exposure (d.p.e). Overall mortality of control mosquitoes was 4.66% (n = 319/6846) at one d.p.e. and 22.5% (n = 1543/6846) at five d.p.e. This was slightly elevated among PIRK exposed mosquitoes at one d.p.e, 6.48% (n = 391/6036) and five d.p.e. 30.7% (n = 1856/6036).

Among host-seeking mosquitoes, control mortality was 4.40% at one d.p.e. (n = 273/6201) and 18.6% at five d.p.e. (n = 1155/6201) and was again slightly higher in the PIRK exposed mosquitoes: 6.07% (n = 262/4319) and 24.6% (n = 1061/4319), respectively. Conversely, mortality of deterred mosquitoes in control chambers was 7.13% after one d.p.e. (n = 46/645) followed by 60.2% at five d.p.e. (n = 388/645), compared to 7.51% (n = 129/1717) and 46.3% (n = 795/1717) at each time point in PIRK chambers (Fig 6).

Fig 6. Risk ratios of secondary outcomes to PIRK exposure. Y axes represent the risk ratio of each outcome for PIRK exposed mosquitoes compared to control chambers. Ratios were calculated based on the age category of the PIRK devices separated on the X axis by color and symbol and denoted in the figure legend. The dotted horizontal line represents risk ratio of 1, or no change between PIRK exposure and control. Numbers greater than one indicate outcomes which are more common in PIRK chambers compared to controls, while numbers lower than one indicate outcomes which were lessened in PIRK chambers.

PIRK exposure was associated with increased mortality at one day (OR: 2.42 [1.43 – 4.09, p = 0.001) and five d.p.e. (OR: 1.50 [1.15 – 1.96], p = 0.003) compared to controls. Higher temperature during the night of capture was associated with slightly higher mortality after one day (scaled temperature OR: 1.25 [1.01 – 1.55], p = 0.043) and five days (OR: 1.17 [1.02 – 1.33], p = 0.025). The deterred population of mosquitoes experienced similar mortality compared to the host-seeking population after 24 hours and increased mortality at five d.p.e. (OR: 4.54 [1.58 – 13.02], p = 0.005). The interaction between population and treatment was borderline significant after five days (OR: 0.34 [0.11 – 1.01], p = 0.053). This interaction term can be interpreted as the effect of PIRK on mortality of deterred mosquitoes relative to the overall effect of PIRK on all mosquito delayed mortality. Caged mosquito density was included as a predictor in all models, with increased density nearing a significant association with decreased mortality at 5 d.p.e. (OR: 0.83 [0.69 – 1.01], p = 0.057). This effect persisted specifically with PIRK-exposed mosquitoes, where higher cage densities were associated with decreased mortality at 1 d.p.e. (OR: 0.38 [0.23 – 0.64], p < 0.001) and 5 d.p.e. (OR: 0.69 [0.52 – 0.90], p = 0.007). Full model coefficients are provided in Table S2.

Blood feeding behavior of host-seeking mosquitoes

Blood feeding rates of host-seeking mosquitoes were measured 12-24 hours post exposure and were slightly lower in PIRK exposed mosquitoes during experimental nights which used freshly opened PIRKs (83.0% compared to 95.8% in control mosquitoes; Fig 6). Blood feeding rates were not reduced in PIRK-exposed mosquitoes at other time points (94.8% in PIRK chambers, 92.6% in control), and model outputs indicate that PIRK exposed mosquitoes blood fed at a reduced rate compared to controls (RR: 0.92 [0.87 – 0.98], p = 0.013) (Table S2). Models estimate this effect diminishes after PIRK aging (weekly "PIRK age" RR: 1.02 [1.00 – 1.05]), and the raw data reveals no impact on blood feeding rates at one week after opening (97.1% feeding rate in mosquitoes from PIRK chambers, 95.3% in control).

Fecundity of blood fed female mosquitoes

Egg laying rates were higher among blood fed females from PIRK chambers (median 8.4 eggs/mosquito, IQR: [6.1 – 14.7]) compared to individuals from control chambers (5.9 eggs/mosquito [4.8 – 10.2]) (Fig 6). Adult cage densities varied between groups (mean number of mosquitoes per control cage: 186; PIRK: 125), with models indicating that the

difference between egg-laying rates was significantly impacted by the adult cage density (RR: 0.61 [0.60 – 0.63], $p < 0.001$) and not overall PIRK status (RR: 0.97 [0.92 – 1.02]). The egg hatch rate was similar between the eggs of control (median hatch rate: 0.52, IQR: [0.45 – 0.60]) and PIRK-exposed mosquitoes (median hatch rate: 0.54, IQR [0.47 – 0.67]) (RR: 1.39 [0.91 – 2.12], $p = 0.13$).

Discussion

The endpoints collected in this trial were designed to measure the personal protection offered by the Personal Insect Repellent Kit (PIRK) and other possible effects on *Anopheles gambiae* vectors that could provide community protection if applied at scale. In addition to landing rates measured by HLC, which have been significantly reduced by transfluthrin-based interventions in prior studies (24,35,36), an additional endpoint – deterrence – was measured by morning outdoor capture of living mosquitoes that were not captured throughout the night. These fates of non-host-seeking mosquitoes in the presence of PIRK – and similar devices – are relevant in a field setting, with deterred mosquitoes possibly diverted to surrounding unprotected households in a manner that may be dose-dependent (26,32).The results from these semi-field trials of PIRK indicate that PIRK is associated with a reduction of approximately 35-40% in overnight mosquito host-seeking behavior in chambers with freshly opened devices, with the effect declining slightly over time but persisting through the testing period lasting five weeks, with additional effects of increased deterrence and mortality compared to unexposed mosquitoes. Host-seeking reduction was observed at all time points, providing evidence for efficacy up to five weeks and suggesting possible efficacy beyond that period. Mosquito mortality was most strongly associated with fresh PIRK devices and mortality trended towards deterrence as the devices aged, possibly related to a dose response as the remaining transfluthrin in the devices declined. The intervention appeared to have little lasting impact on disarming blood feeding behavior, fecundity, or fertility. These results indicate that the intervention functions as expected through the primary mode of action in reducing landing, but the impacts are not limited to reduction in landing and the impact on disease transmission may be considered based on the accumulation of these effects.

It is possible that host-seeking behavior is over-estimated in this study, as the closed semi-field design forces non-host-seeking mosquitoes to remain within 10 meters of host-seeking cues from the human landing collectors

throughout the night. In control chambers, hourly landing rates were higher in the first hour than all other hours, highlighting an hourly difference in host-seeking avidity that may be an artifact of the semi-field setting, considering that the natural circadian rhythms of *An. gambiae s.s.* generally peak after midnight. In models which consider the hourly HLC recapture rate, the predicted effect of PIRK is elevated roughly 50% above the observed all-night reduction of host-seeking, driven by the large difference in activity in the first hour which then "trails off" throughout the night (Fig 4). Maximum response in the first hour has been observed in other semi field studies (37,38), and some authors have used multiple releases throughout the night to maintain mosquito biting pressure (39). This straightforward change to semi-field design should be considered in future designs to investigate these hourly differences specifically to determine if they are more closely related to mosquito behavior within this closed system or product efficacy. With these considerations, the results of this study can be interpreted with the nightly efficacy acting as a more conservative estimate compared to the hourly results.

The secondary endpoints measured in this experiment were chosen to reflect outcomes of epidemiological importance in the field and from modeling studies (33). Mosquito mortality or reductions in fitness have been observed in prior studies of transfluthrin (27–29), providing a mechanism for community protection through overall suppression of mosquito populations and reduced age structures (40,41). Deterrence and knockdown were both elevated in PIRK chambers and may be dose-dependent, with the ratio of mortality to deterrence highest when testing fresh PIRK devices (Fig 5). Notably, the exposure-related mortality was largely observed in the perimeter ditches of treatment chambers. It is possible these mosquitoes were repelled by the PIRK devices and would have escaped in a natural setting but were prevented from doing so by the confines of the chamber, leading to over-estimation of mortality in this study. Their accumulation in the perimeter ditches also prevented the differentiation between mortality and knockdown effects.

The fates of host-seeking and deterred mosquitoes are also relevant in the context of community protection. In addition to the acute mortality/knockdown which may occur during exposure, delayed impacts on mosquito survival can contribute to community protection. In this study, mortality of host-seeking and deterred mosquitoes was observed for five days. A large increase in mortality was observed among "deterred" mosquitoes in both treated and untreated chambers after five days which was not present at one day, likely driven by the lack of a bloodmeal provided to these mosquitoes at the one-day time point. Mortality was significantly increased in PIRK-exposed HLC-captured mosquitoes

at both time points, but this deleterious effect of PIRK was very nearly significantly reversed among deterred mosquitoes after five days (OR: 0.34 [0.11 – 1.01], p = 0.053). This suggests that deterred mosquitoes could be less negatively impacted by PIRK compared to host-seeking mosquitoes, possibly due to lower exposure of active ingredient outside of the huts. It is also possible that – considering they missed twelve hours of feeding opportunities – the 'deterred' mosquitoes found in control chambers represent a particularly unfit subset of the original population, resulting in abnormally high mortality. This alternative explanation provides further support for a nightly multiple release experimental design. This deterrence effect should be further studied, as deterred mosquitoes appear capable of enduring PIRK exposure and may divert to other nearby hosts. This finding also supports the ability of this semi-field system to estimate repellency and/or deterrence mechanisms, although an idealized design would be substantially larger than the expected area of effect of the tested device.

Prior studies of transfluthrin have utilized proxy measurements for blood feeding such as HLC (42), or allowed mosquitoes to freely bite to measure reductions in blood feeding (28). It has been suggested in *Aedes* mosquitoes that landing and biting inhibition might differ (43), and separating these endpoints allows for host-seeking and probing behaviors to be considered separately. This disarming endpoint measured by a prolonged blood feeding inhibition even after exposure is particularly important to capture in semi-field systems, since biting behavior cannot be well quantified during field trials involving HLC or other trapping methods. In this study, blood feeding rates among host-seeking mosquitoes observed 12-24 hours post exposure were slightly, but significantly depressed in mosquitoes exposed to PIRKs, with the effect observed to entirely diminish by the first week after opening. This reduction in blood feeding behavior appears to be short-lived but should continue to be further studied in the presence or immediate aftermath of PIRK exposure, rather than 12-24 hours post exposure, to measure for how long after exposure mosquitoes are disarmed and if disarmament provides a community effect by delaying feeding cycles (27,33). Following successful feeding, egg-laying and hatch rates were slightly higher among PIRK exposed mosquitoes overall but varied by PIRK age without following a clear trend. Models suggested that both rates were driven by the mosquito density in experimental cages, which was considerably higher among control mosquitoes, rather than PIRK status.

Temperature and humidity also appear to play a role in mosquito behavior and PIRK efficacy, with higher nightly temperature and humidity associated with reduced PIRK efficacy in these experiments. In hourly analysis, higher

humidity was associated with generally higher host-seeking across both chambers, while higher temperature and lowered humidity were associated with reduced PIRK efficacy. It is unclear why the association of humidity with PIRK efficacy is reversed in hourly and nightly analysis; it's possible that it is a byproduct of improved model fit due to higher data resolution, or an indication of mosquito behavioral patterns. Overall, these experiments were conducted in cooler than optimal temperatures for volatile pyrethroids with a mean nightly temperature around 21C. Increased temperatures resulting in reduced PIRK efficacy contrasts with other studies that have shown improved efficacy at higher temperatures (26).

Conclusions

The results of this semi-field study suggest that the PIRK device, a passive emanator of the pyrethroid transfluthrin, could be useful as a malaria control tool. Over the course of experimental nights, human landing was reduced at all time points over the five-week observation period after unsealing the devices, with evidence for heightened mortality transitioning towards deterrence effects over the use of the intervention. Landing rates were reduced up to and including five weeks past opening, with further duration of effect unknown from these trials. Overall rates of deterrence and mortality decreased over the five-week period in addition to the trend towards deterrence, suggesting a possible dose response. In general, there was very little impact on disarming, fecundity, or fertility, except in the case of fresh PIRKs where there was a small but significant decrease in blood feeding (a disarming effect). The data gathered in this study also support possible improvements for semi-field experimental design, primarily shorter recapture timeframes and repeated releases during experimental nights to better mimic a field environment where approaching mosquitoes are more consistently naïve to the active ingredient being tested. We also recommend that temperature and humidity is always monitored when evaluating the efficacy of personal protection tools.

Acknowledgements:

The authors would like to acknowledge the Macha Research Trust entomology staff who served as mosquito collectors and provided insectary support for the project. Additionally, the MRT administrative and support staff for various vital contributions.

References

1. Bhatt S, Weiss DJ, Cameron E, Bisanzio D, Mappin B, Dalrymple U, et al. The effect of malaria control on *Plasmodium falciparum* in Africa between 2000 and 2015. Nature. 2015;526:207–11.

2. Bayoh MN, Mathias DK, Odiere MR, Mutuku FM, Kamau L, Gimnig JE, et al. Anopheles gambiae : historical population decline associated with regional distribution of insecticide-treated bed nets in western Nyanza Province , Kenya. 2010;1–12.

3. Mccann RS, Ochomo E, Bayoh MN, Vulule JM, Hamel MJ, Gimnig JE, et al. Reemergence of Anopheles funestus as a Vector of Plasmodium falciparum in Western Kenya after Long-Term Implementation of Insecticide-Treated Bed Nets. 2014;90(4):597–604.

4. Cooke MK, Kahindi SC, Oriango RM, Owaga C, Ayoma E, Mabuka D, et al. “A bite before bed”: Exposure to malaria vectors outside the times of net use in the highlands of western Kenya. Malar J. 2015;14(1):1–15.

5. Stevenson J, St. Laurent B, Lobo NF, Cooke MK, Kahindi SC, Oriango RM, et al. Novel Vectors of Malaria Parasites in the Western Highlands of Kenya. Emerg Infect Dis. 2012;18(9):1547–9.

6. Lobo NF, Laurent B St., Sikaala CH, Hamainza B, Chanda J, Chinula D, et al. Unexpected diversity of *Anopheles* species in Eastern Zambia: implications for evaluating vector behavior and interventions using molecular tools. Sci Rep. 2015;5:17952.

7. Laurent BS, Cooke M, Krishnankutty SM, Asih P, Mueller JD, Kahindi S, et al. Molecular characterization reveals diverse and unknown malaria vectors in the western Kenyan highlands. Am J Trop Med Hyg. 2016;94(2):327–35.

8. Sherrard-Smith E, Skarp JE, Beale AD, Fornadel C, Norris LC, Moore SJ, et al. Mosquito feeding behavior and how it influences residual malaria transmission across Africa. Proc Natl Acad Sci U S A. 2019;116(30):15086–96.

9. Bayoh MN, Walker ED, Kosgei J, Ombok M, Olang GB, Githeko AK, et al. Persistently high estimates of late night , indoor exposure to malaria vectors despite high coverage of insecticide treated nets. 2014;1–13.

10. Lwetoijera DW, Harris C, Kiware SS, Dongus S, Devine GJ, Mccall PJ, et al. Increasing role of Anopheles funestus

and Anopheles arabiensis in malaria transmission in the Kilombero Valley , Tanzania. 2014;1–10.

11. Moiroux N, Damien GB, Egrot M, Djenontin A, Chandre F, Corbel V, et al. Human exposure to early morning Anopheles funestus biting behavior and personal protection provided by long-lasting insecticidal nets. PLoS One. 2014;9(8):8–11.

12. Kibret S, Wilson GG. Increased outdoor biting tendency of Anopheles arabiensis and its challenge for malaria control in Central Ethiopia. Public Health [Internet]. 2016;141(November 2012):143–5. Available from: http://dx.doi.org/10.1016/j.puhe.2016.09.012

13. Thomsen EK, Koimbu G, Pulford J, Jamea-Maiasa S, Ura Y, Keven JB, et al. Mosquito behavior change after distribution of bednets results in decreased protection against malaria exposure. J Infect Dis. 2017;215(5):790–7.

14. Musiime AK, Smith DL, Kilama M, Rek J, Arinaitwe E, Nankabirwa JI, et al. Impact of vector control interventions on malaria transmission intensity, outdoor vector biting rates and Anopheles mosquito species composition in Tororo, Uganda. Malar J [Internet]. 2019;18(1):1–9. Available from: https://doi.org/10.1186/s12936-019-3076-4

15. Hawley WA, Phillips-howard PA, Kuile FOTER, Terlouw DJ, Vulule JM, Ombok M, et al. Community-wide effects of permethrin-treated bed nets on child mortality and malaria morbidity in western Kenya. 2003;68(Suppl 4):121–7.

16. Killeen GF, Smith TA, Ferguson HM, Mshinda H, Abdulla S, Lengeler C, et al. Preventing childhood malaria in Africa by protecting adults from mosquitoes with insecticide-treated nets. PLoS Med. 2007;4(7):1246–58.

17. Strode C, Donegan S, Garner P, Enayati AA, Hemingway J. The Impact of Pyrethroid Resistance on the Efficacy of Insecticide-Treated Bed Nets against African Anopheline Mosquitoes: Systematic Review and Meta-Analysis. PLoS Med. 2014;11(3).

18. Churcher TS, Lissenden N, Griffin JT, Worrall E, Ranson H. The impact of pyrethroid resistance on the efficacy and effectiveness of bednets for malaria control in Africa. Elife. 2016;5(AUGUST):1–26.

19. Mosha JF, Kulkarni MA, Lukole E, Matowo NS, Pitt C, Messenger LA, et al. Articles Effectiveness and cost-effectiveness against malaria of three types of dual-active-ingredient long-lasting insecticidal nets (LLINs) compared with pyrethroid-only LLINs in Tanzania : a four-arm , cluster-randomised trial. Lancet [Internet].

2022;399(10331):1227–41. Available from: http://dx.doi.org/10.1016/S0140-6736(21)02499-5

20. WHO Guidelines for malaria, 3 June 2022. Geneva: World Health Organization; 2022.

21. Tusting LS, Bottomley C, Gibson H, Kleinschmidt I, Tatem AJ, Lindsay SW, et al. Housing Improvements and Malaria Risk in Sub-Saharan Africa: A Multi-Country Analysis of Survey Data. PLoS Med. 2017;14(2):1–15.

22. Killeen GF, Tatarsky A, Diabate A, Chaccour CJ, Marshall JM, Okumu FO, et al. Developing an expanded vector control toolbox for malaria elimination. BMJ Glob Heal. 2017;2(2):1–8.

23. Monroe A, Mihayo K, Okumu F, Finda M, Moore S, Koenker H, et al. Human behaviour and residual malaria transmission in Zanzibar: Findings from in-depth interviews and direct observation of community events. Malar J [Internet]. 2019;18(1):1–13. Available from: https://doi.org/10.1186/s12936-019-2855-2

24. Masalu JP, Finda M, Okumu FO, Minja EG, Mmbando AS, Sikulu-Lord MT, et al. Efficacy and user acceptability of transfluthrin-treated sisal and hessian decorations for protecting against mosquito bites in outdoor bars. Parasites and Vectors. 2017;10(1):1–8.

25. Mmbando AS, Ngowo H, Limwagu A, Kilalangongono M, Kifungo K, Okumu FO. Eave ribbons treated with the spatial repellent, transfluthrin, can effectively protect against indoor-biting and outdoor-biting malaria mosquitoes. Malar J [Internet]. 2018;17(1):1–14. Available from: https://doi.org/10.1186/s12936-018-2520-1

26. Ogoma SB, Mmando AS, Swai JK, Horstmann S, Malone D, Killeen GF. A low technology emanator treated with the volatile pyrethroid transfluthrin confers long term protection against outdoor biting vectors of lymphatic filariasis, arboviruses and malaria. PLoS Negl Trop Dis. 2017;11(4).

27. Ogoma SB, Ngonyani H, Simfukwe ET, Mseka A, Moore J, Maia MF, et al. The mode of action of spatial repellents and their impact on vectorial capacity of Anopheles gambiae sensu stricto. PLoS One. 2014;9(12):1–21.

28. Ogoma SB, Lorenz LM, Ngonyani H, Sangusangu R, Kitumbukile M, Kilalangongono M, et al. An experimental hut study to quantify the effect of DDT and airborne pyrethroids on entomological parameters of malaria transmission. Malar J [Internet]. 2014;13(1):1–17. Available from: Malaria Journal

29. Tambwe MM, Mbeyela EM, Massinda BM, Moore SJ, Maia MF. Experimental hut evaluation of linalool spatial repellent agar gel against Anopheles gambiae sensu stricto mosquitoes in a semi-field system in Bagamoyo, Tanzania. Parasites and Vectors. 2014;7(1):1–6.

30. Bibbs CS, Tsikolia M, Bloomquist JR, Bernier UR, Xue R De, Kaufman PE. Vapor toxicity of five volatile pyrethroids against Aedes aegypti, Aedes albopictus, Culex quinquefasciatus, and Anopheles quadrimaculatus (Diptera: Culicidae). Pest Manag Sci. 2018;74(12):2699–706.

31. Mwanga EP, Mmbando AS, Mrosso PC, Stica C, Mapua SA, Finda MF, et al. Eave ribbons treated with transfluthrin can protect both users and non-users against malaria vectors. Malar J [Internet]. 2019;18(1):1–14. Available from: https://doi.org/10.1186/s12936-019-2958-9

32. Maia MF, Kreppel K, Mbeyela E, Roman D, Mayagaya V, Lobo NF, et al. A crossover study to evaluate the diversion of malaria vectors in a community with incomplete coverage of spatial repellents in the Kilombero Valley, Tanzania. Parasites and Vectors [Internet]. 2016;9(1):1–13. Available from: http://dx.doi.org/10.1186/s13071-016-1738-4

33. Denz A, Njoroge MM, Tambwe MM, Champagne C, Okumu F, van Loon JJA, et al. Predicting the impact of outdoor vector control interventions on malaria transmission intensity from semi-field studies. Parasites and Vectors [Internet]. 2021;14(1):1–22. Available from: https://doi.org/10.1186/s13071-020-04560-x

34. Silver JB. Mosquito Ecology: Field sampling methods [Internet]. 3rd ed. Springer Netherlands; 2008. 1477 p. Available from: 10.1007/978-1-4020-6666-5

35. Ogoma SB, Ngonyani H, Simfukwe ET, Mseka A, Moore J, Killeen GF. Spatial repellency of transfluthrin-treated hessian strips against laboratory-reared Anopheles arabiensis mosquitoes in a semi-field tunnel cage. Parasites and Vectors [Internet]. 2012;5(1):54. Available from: http://www.parasitesandvectors.com/content/5/1/54

36. Govella NJ, Ogoma SB, Paliga J, Chaki PP, Killeen G. Impregnating hessian strips with the volatile pyrethroid transfluthrin prevents outdoor exposure to vectors of malaria and lymphatic filariasis in urban Dar es Salaam, Tanzania. Parasites and Vectors [Internet]. 2015;8(1):8–12. Available from: http://dx.doi.org/10.1186/s13071-

015-0937-8

37. Sangoro O, Lweitojera D, Simfukwe E, Ngonyani H, Mbeyela E, Lugiko D, et al. Use of a semi-field system to evaluate the efficacy of topical repellents under user conditions provides a disease exposure free technique comparable with field data. 2014;1–11.

38. Milali MP, Lord MTS, Govella NJ. Bites before and after bedtime can carry a high risk of human malaria infection. Malar J. 2017;1–10.

39. Mbuba E, Odufuwa OG, Tenywa FC, Philipo R, Tambwe MM, Swai JK, et al. Single blinded semi ‑ field evaluation of MAÏA ® topical repellent ointment compared to unformulated 20 % DEET against Anopheles gambiae , Anopheles arabiensis and Aedes aegypti in Tanzania. Malar J [Internet]. 2021;1–10. Available from: https://doi.org/10.1186/s12936-020-03461-9

40. Magesa SM, Wilkes TJ, Mnzava AEP, Njunwa KJ, Myamba J, Kivuyo MDP, et al. Trial of pyrethroid impregnated bednets in an area of Tanzania holoendemic for malaria Part 2 . Effects on the malaria vector population. 1991;49:97–108.

41. Killeen GF, Smith TA. Exploring the contributions of bed nets , cattle , insecticides and excitorepellency to malaria control : a deterministic model of mosquito host-seeking behaviour and mortality. 2007;

42. Anaele BI, Varshney K, Ugwu FSO, Frasso R. The efficacy of insecticide-treated window screens and eaves against Anopheles mosquitoes: a scoping review. Malar J. 2021;20(1):1–18.

43. Lucas JR, Shono Y, Iwasaki T, Ishiwatari T, Spero N, Benzon G. U.S. Laboratory and field trials of metofluthrin (SumiOne®) emanators for reducing mosquito biting outdoors. J Am Mosq Control Assoc. 2007;23(1):47–54.

Supporting information

S1 Fig. Nightly temperature and humidity mean values over the course of experiments in the semi-field system. Measurements were taken from a weather station (Onset HOBO) adjacent to the semi-field enclosure. Values are plotted on the same axis, with humidity reported as relative humidity (percentage) and temperature reported in Celsius.

Table S1. Model coefficients for primary experimental outcomes. All models were mixed effect generalized linear models (GLMER) with a Poisson (log) link function. Each model included the log-transformed number of mosquitoes released into the chamber as the exposure term, except for the hourly host-seeking model which uses the log-transformed number of mosquitoes remaining in the chamber at each hour. Models were assessed by AIC and coefficients which were dropped to enable model convergence are denoted with a dash '-'. AIC and degrees of freedom for the null model are displayed in parenthesis after the values for each fitted model. Coefficients which are not relevant to a specific model are denoted with an NA. Date of experiment was included in all models as a random effect. P values are coded, with '***' representing p values < 0.001, '**' representing p values between 0.001 and 0.01, '*' between 0.01 and 0.05, and '.' representing nearly significant p values between 0.05 and 0.1. [&]Temperature and Humidity were centered and scaled around their mean values for all models.

Table S2. Model coefficients for delayed mortality and blood feeding behavior. All models were mixed effect generalized linear models (GLMER) with a binomial (logit) link function. Models were assessed by AIC and coefficients which were dropped to enable model convergence are denoted with a dash '-'. AIC and degrees of freedom for the null model are displayed in parenthesis after the values for each fitted model. Coefficients which are not relevant to a specific model are denoted with an NA. Date of experiment was included in all models as a random effect. P values are coded, with '***' representing p values < 0.001, '**' representing p values between 0.001 and 0.01, '*' between 0.01 and 0.05, and '.' representing nearly significant p values between 0.05 and 0.1. [&]Coefficients denoted with this symbol were centered and scaled around their mean values prior to model fitting. [%]The age of treatment was considered as a numeric predictor in all models except the blood feeding model, where a binary factor (fresh vs not fresh) was used instead to better model the observed behavior.

Fig 1

Click here to access/download;Figure;Fig1.tif

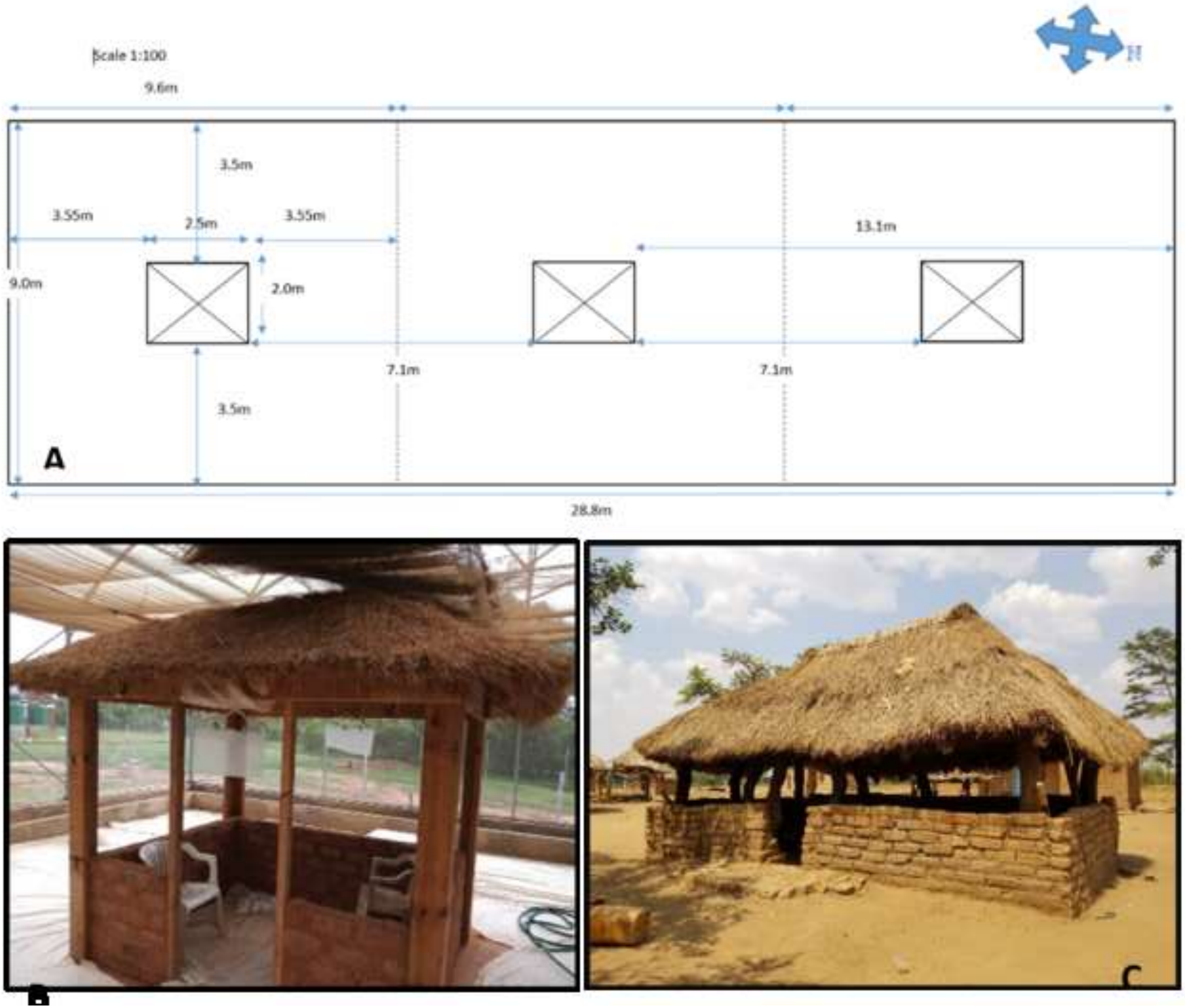

Fig 2

Click here to access/download;Figure;Fig2.tif

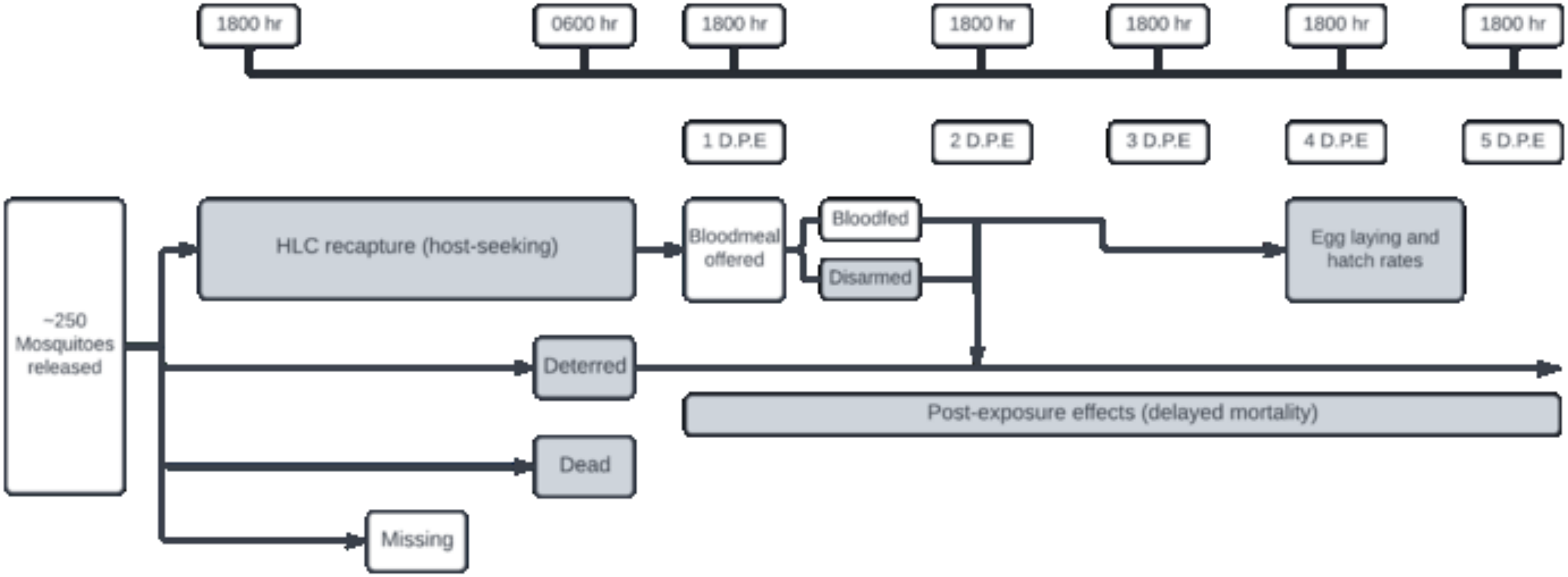

Fig 3

Click here to access/download;Figure;Fig3.tiff

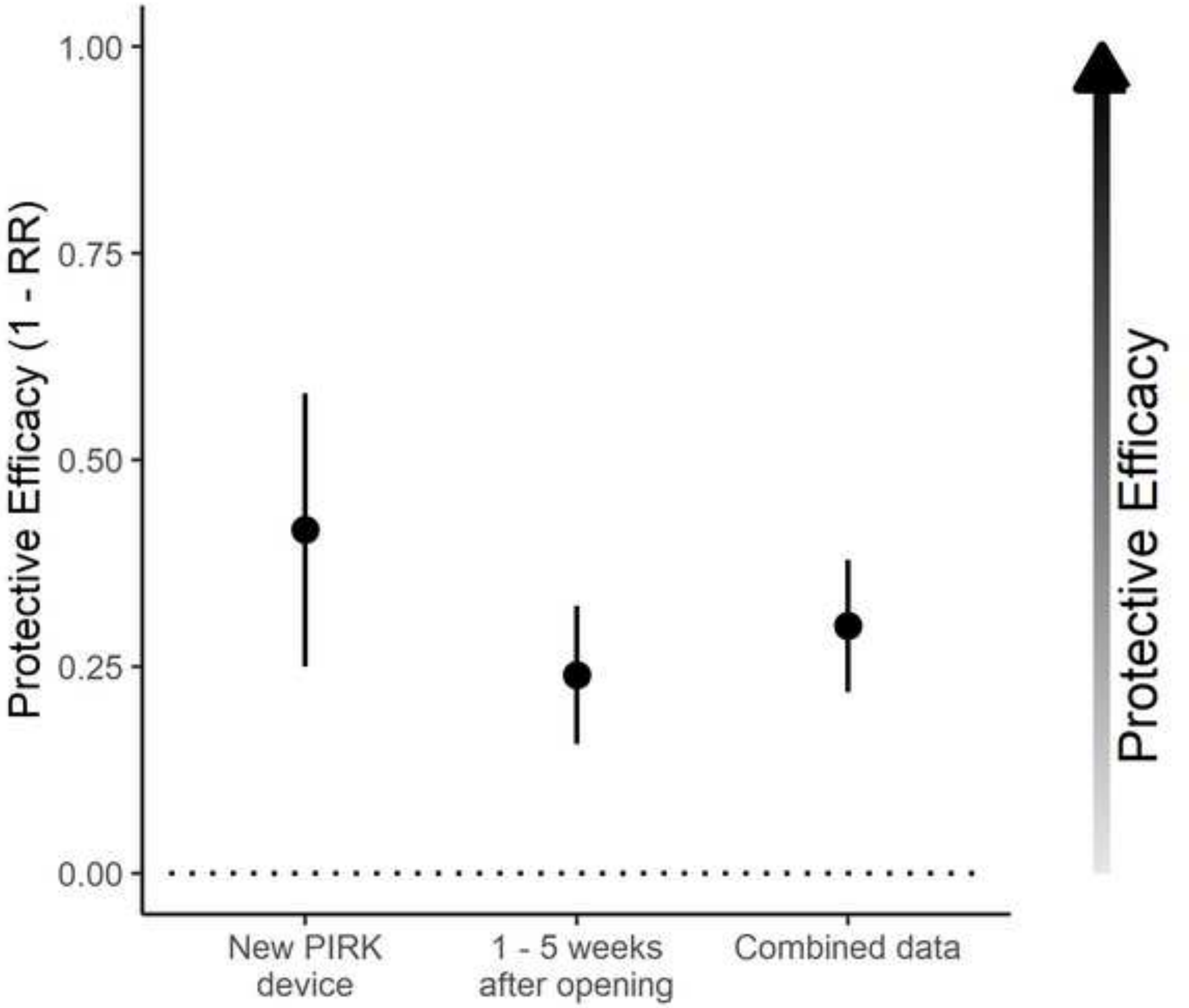

Fig 4

Click here to access/download;Figure;Fig4.tiff

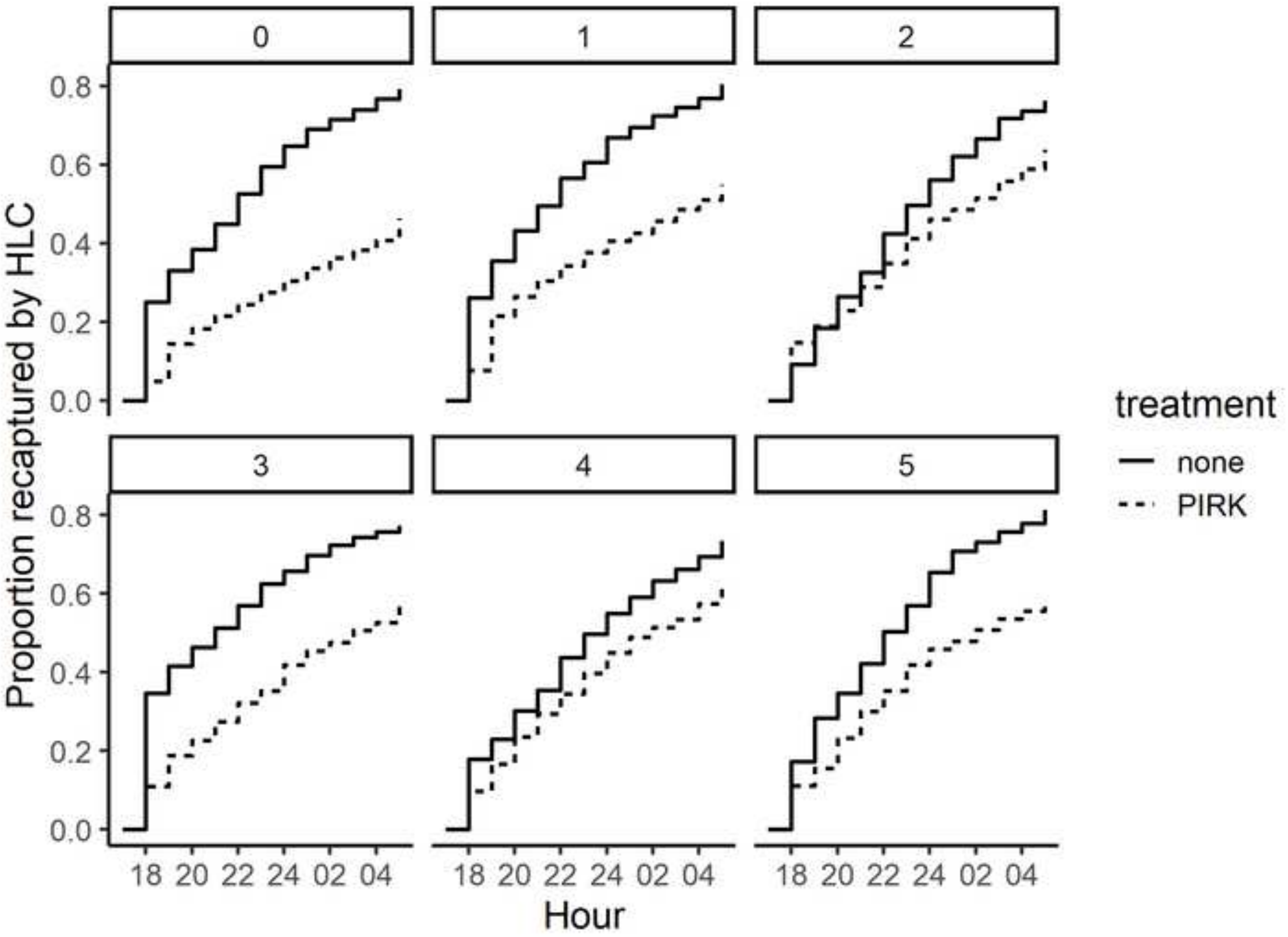

Fig 5

Click here to access/download;Figure;Fig5.tiff

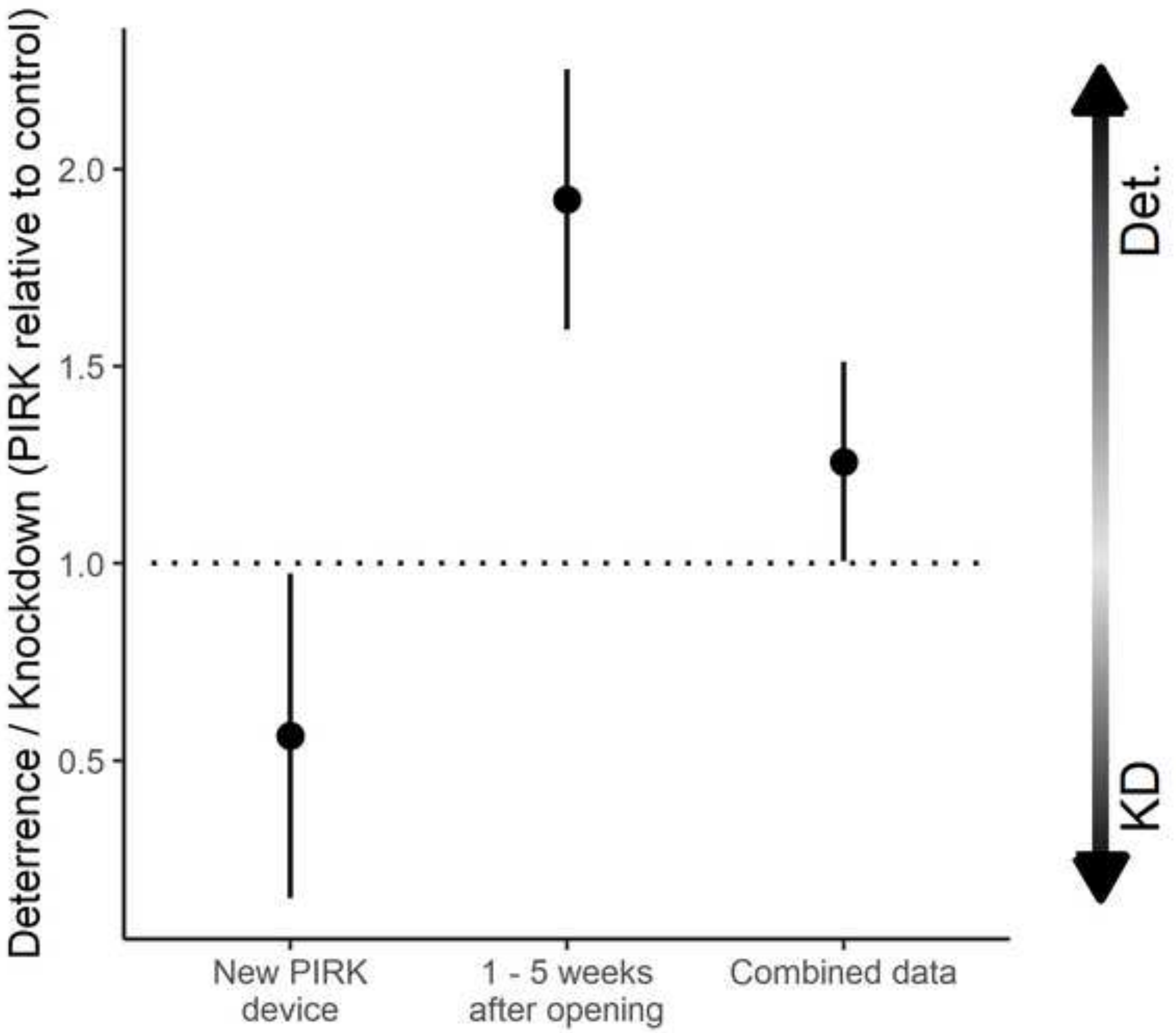

Fig 6

Click here to access/download;Figure;Fig6.tiff

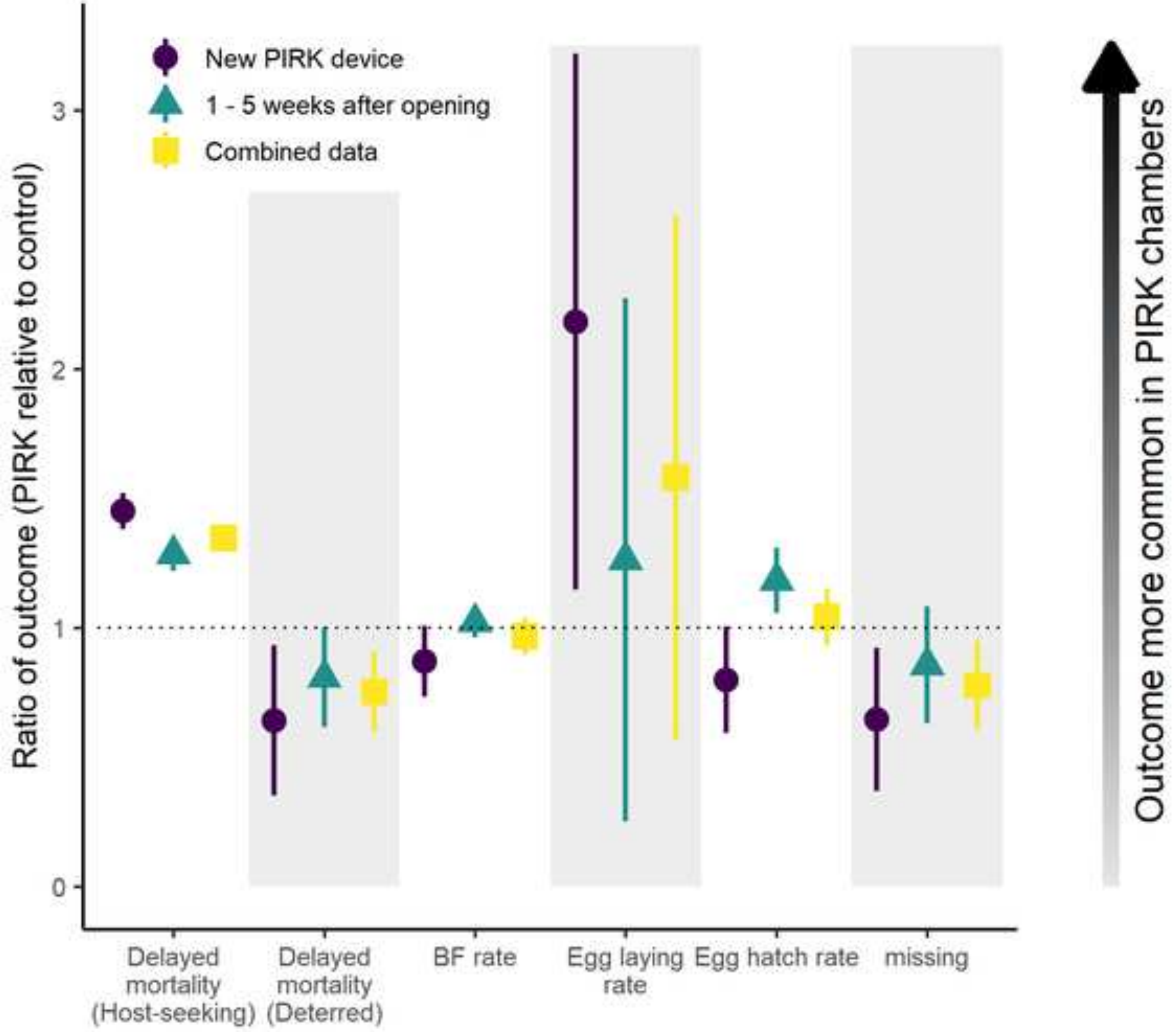

Fig S1

Click here to access/download
Supporting Information
FigS1.tiff

Table S1

Click here to access/download
Supporting Information
TableS1.png

Table S2

Click here to access/download
Supporting Information
TableS2.png

Click here to access/download
Supporting Information
PIRK_SemiField_SOP.docx
