# Supplementary material for: Semi-field evaluation of a volatile transfluthrin-based intervention reveals efficacy as a spatial repellent and evidence of other modes of action
Source: PLoS One. 2023 May 11;18(5):e0285501. doi: 10.1371/journal.pone.0285501 (PMC10174509; doi:10.1371/journal.pone.0285501)
Supplement: Attachment — Submitted filename: Response to Reviewers.docx [file pone.0285501.s007.docx]

Dziedzom Komi de Souza, PhD

*Academic Editor*

PLOS ONE

November 22, 2022

Dear Dr. de Souza,

We would like to thank you and the editors for the time in reviewing our manuscript. We have addressed the comments made by the editor and reviewer in our revised manuscript attached. Additionally, we were requested by the manufacturer to refrain from referring to the product as we had been earlier (as “PIRK”). Therefore, additional changes were made to the manuscript, figures, tables, etc. where necessary to change the name to refer more generically to a “volatile pyrethroid based spatial repellent (VPSR) product”.

Specific point feedback:

Editor:

Thank you for taking the time to review our manuscript, and for your flexibility in allowing us to extend our revision deadline. Individual comments are below:

1. I have adjusted the formatting of the manuscript to reflect the PLOS ONE style templates.
2. The questionnaire is included. Most of the local approvals gained during this study were related to the second phase, a field study which is in preparation for submission, while this phase didn’t involve local community members.
3. The funders had no role in study design, data collection and analysis, decision to publish, or preparation of the manuscript.
4. I believe the copyrighted portion of Figure 1 is the floorplan of the semi-field chambers (Fig 1A). I took the opportunity to draft a new floorplan drawing which (if I understand correctly) should not require a copyright license agreement, and improves on the original figure by highlighting more relevant dimensions. The other two photographs are my own personal photographs and have not been published anywhere before.
5. I was not able to add the new references with tracked changes on due to my reference manager, but three additional references (#32-34) were added in the discussion in response to comments from reviewer #2 regarding the efficacy of transfluthrin against mosquitoes resistant to other pyrethroids.

Reviewer #1:

We would like to thank you for your time during your review of our manuscript. in response to your point about using a susceptible mosquito population alone, we agree that it is a limitation. We were unable to use a resistant strain since we didn’t have an available colony. We have added text to the manuscript to clarify that, and an additional paragraph in the discussion related to this and the similar limitation of using *Anopheles gambiae* when other species often exhibit the behaviors of interest (outdoor biting, etc.)

We agree and have incorporated each of your individual edits into the revised manuscript.

- Regarding starving duration, mosquitoes were starved for four hours. This has been clarified in the text.
- The release and start times both being listed as 18:00 was a mistake that has been corrected. The mosquitoes were released in the chambers at 17:30 and the collectors would enter the chambers and start at 18:00.
- The denominators in the text were a remnant from a previous draft where those numbers (e.g. 7974) had been discussed in the previous paragraph regarding recovery rates. The denominators referred to the total number of mosquitoes recovered from the experiments (excluding those that were missing/never recovered). They have since been amended, and the denominators now refer to the total number of mosquitoes released into the chambers. This also better reflects how the models were constructed, using the total release # as the offset in the Poisson models.

Reviewer #2:

We would like to thank you for the time taken during your consideration of our manuscript. We have incorporated your editorial suggestions into the revised manuscript and have included a paragraph in the discussion addressing your important points about *An. arabiensis* and the effect of transfluthrin on pyrethroid species. In brief, we agree regarding the behavior of the two (and other) vector species, and transfluthrin has been shown to inhibit pyrethroid-resistant species in the past, but there is no published data regarding resistance of mosquitoes towards transfluthrin or development of transfluthrin resistance in the field or lab.

Sincerely,

Tim Burton, PhD candidate

University of Notre Dame

*Department of Biological Sciences*

Notre Dame, IN 46556
