# Supplementary material for: Semi-field evaluation of a volatile transfluthrin-based intervention reveals efficacy as a spatial repellent and evidence of other modes of action
Source: PLoS One. 2023 May 11;18(5):e0285501. doi: 10.1371/journal.pone.0285501 (PMC10174509; doi:10.1371/journal.pone.0285501)
Supplement: Peer review history

# Peer review: Decision Letter 0

**DOI:** 10.1371/journal.pone.0285501.r001

**Authors:** Dziedzom Komi de Souza

2 Feb 2023

PONE-D-22-32222Semi-field evaluation of a volatile transfluthrin based intervention reveals efficacy as a spatial repellent and evidence of other modes of actionPLOS ONE

Dear Dr. Burton,

Thank you for submitting your manuscript to PLOS ONE. After careful consideration, we feel that it has merit but does not fully meet PLOS ONE’s publication criteria as it currently stands. Therefore, we invite you to submit a revised version of the manuscript that addresses the points raised during the review process.

Please submit your revised manuscript by Mar 19 2023 11:59PM. If you will need more time than this to complete your revisions, please reply to this message or contact the journal office at plosone@plos.org. When you're ready to submit your revision, log on to https://www.editorialmanager.com/pone/ and select the 'Submissions Needing Revision' folder to locate your manuscript file.

Please include the following items when submitting your revised manuscript:

- A rebuttal letter that responds to each point raised by the academic editor and reviewer(s). You should upload this letter as a separate file labeled 'Response to Reviewers'.
- A marked-up copy of your manuscript that highlights changes made to the original version. You should upload this as a separate file labeled 'Revised Manuscript with Track Changes'.
- An unmarked version of your revised paper without tracked changes. You should upload this as a separate file labeled 'Manuscript'.

If you would like to make changes to your financial disclosure, please include your updated statement in your cover letter. Guidelines for resubmitting your figure files are available below the reviewer comments at the end of this letter.

If applicable, we recommend that you deposit your laboratory protocols in protocols.io to enhance the reproducibility of your results. Protocols.io assigns your protocol its own identifier (DOI) so that it can be cited independently in the future. For instructions see: https://journals.plos.org/plosone/s/submission-guidelines#loc-laboratory-protocols. Additionally, PLOS ONE offers an option for publishing peer-reviewed Lab Protocol articles, which describe protocols hosted on protocols.io. Read more information on sharing protocols at https://plos.org/protocols?utm_medium=editorial-email&utm_source=authorletters&utm_campaign=protocols.

We look forward to receiving your revised manuscript.

Kind regards,

Dziedzom Komi de Souza, Ph.D.

Academic Editor

PLOS ONE

Journal Requirements:

When submitting your revision, we need you to address these additional requirements.

1. Please ensure that your manuscript meets PLOS ONE's style requirements, including those for file naming. The PLOS ONE style templates can be found at 

https://journals.plos.org/plosone/s/file?id=wjVg/PLOSOne_formatting_sample_main_body.pdf and https://journals.plos.org/plosone/s/file?id=ba62/PLOSOne_formatting_sample_title_authors_affiliations.pdf

2. Please include a complete copy of PLOS’ questionnaire on inclusivity in global research in your revised manuscript. Our policy for research in this area aims to improve transparency in the reporting of research performed outside of researchers’ own country or community. The policy applies to researchers who have travelled to a different country to conduct research, research with Indigenous populations or their lands, and research on cultural artefacts. The questionnaire can also be requested at the journal’s discretion for any other submissions, even if these conditions are not met.  Please find more information on the policy and a link to download a blank copy of the questionnaire here: https://journals.plos.org/plosone/s/best-practices-in-research-reporting. Please upload a completed version of your questionnaire as Supporting Information when you resubmit your manuscript.

3. Thank you for stating the following financial disclosure: 

"This study was sponsored by the Department of the Army, U.S. Army Contracting Command, Aberdeen Proving Ground, Edgewood Contracting Division, Ft Detrick MD [under Deployed Warfighter Protection (DWFP) Program Grant #W911QY1810001]."

Please state what role the funders took in the study. If the funders had no role, please state: ""The funders had no role in study design, data collection and analysis, decision to publish, or preparation of the manuscript."" 

If this statement is not correct you must amend it as needed. 

Please include this amended Role of Funder statement in your cover letter; we will change the online submission form on your behalf.

4. We note that Figure 1 in your submission contain copyrighted images. All PLOS content is published under the Creative Commons Attribution License (CC BY 4.0), which means that the manuscript, images, and Supporting Information files will be freely available online, and any third party is permitted to access, download, copy, distribute, and use these materials in any way, even commercially, with proper attribution. For more information, see our copyright guidelines: http://journals.plos.org/plosone/s/licenses-and-copyright.

We require you to either (1) present written permission from the copyright holder to publish these figures specifically under the CC BY 4.0 license, or (2) remove the figures from your submission:

a. You may seek permission from the original copyright holder of Figure 1 to publish the content specifically under the CC BY 4.0 license. 

We recommend that you contact the original copyright holder with the Content Permission Form (http://journals.plos.org/plosone/s/file?id=7c09/content-permission-form.pdf) and the following text:

“I request permission for the open-access journal PLOS ONE to publish XXX under the Creative Commons Attribution License (CCAL) CC BY 4.0 (http://creativecommons.org/licenses/by/4.0/). Please be aware that this license allows unrestricted use and distribution, even commercially, by third parties. Please reply and provide explicit written permission to publish XXX under a CC BY license and complete the attached form.”

Please upload the completed Content Permission Form or other proof of granted permissions as an ""Other"" file with your submission. 

In the figure caption of the copyrighted figure, please include the following text: “Reprinted from [ref] under a CC BY license, with permission from [name of publisher], original copyright [original copyright year].”

b. If you are unable to obtain permission from the original copyright holder to publish these figures under the CC BY 4.0 license or if the copyright holder’s requirements are incompatible with the CC BY 4.0 license, please either i) remove the figure or ii) supply a replacement figure that complies with the CC BY 4.0 license. Please check copyright information on all replacement figures and update the figure caption with source information. If applicable, please specify in the figure caption text when a figure is similar but not identical to the original image and is therefore for illustrative purposes only.

5. Please review your reference list to ensure that it is complete and correct. If you have cited papers that have been retracted, please include the rationale for doing so in the manuscript text, or remove these references and replace them with relevant current references. Any changes to the reference list should be mentioned in the rebuttal letter that accompanies your revised manuscript. If you need to cite a retracted article, indicate the article’s retracted status in the References list and also include a citation and full reference for the retraction notice.

[Note: HTML markup is below. Please do not edit.]

Reviewers' comments:

Reviewer's Responses to Questions

**Comments to the Author**

1. Is the manuscript technically sound, and do the data support the conclusions?

The manuscript must describe a technically sound piece of scientific research with data that supports the conclusions. Experiments must have been conducted rigorously, with appropriate controls, replication, and sample sizes. The conclusions must be drawn appropriately based on the data presented. 

Reviewer #1: Yes

Reviewer #2: Yes

**********

2. Has the statistical analysis been performed appropriately and rigorously? 

Reviewer #1: Yes

Reviewer #2: Yes

**********

3. Have the authors made all data underlying the findings in their manuscript fully available?

The PLOS Data policy requires authors to make all data underlying the findings described in their manuscript fully available without restriction, with rare exception (please refer to the Data Availability Statement in the manuscript PDF file). The data should be provided as part of the manuscript or its supporting information, or deposited to a public repository. For example, in addition to summary statistics, the data points behind means, medians and variance measures should be available. If there are restrictions on publicly sharing data—e.g. participant privacy or use of data from a third party—those must be specified.

Reviewer #1: Yes

Reviewer #2: Yes

**********

4. Is the manuscript presented in an intelligible fashion and written in standard English?

PLOS ONE does not copyedit accepted manuscripts, so the language in submitted articles must be clear, correct, and unambiguous. Any typographical or grammatical errors should be corrected at revision, so please note any specific errors here.

Reviewer #1: Yes

Reviewer #2: Yes

**********

5. Review Comments to the Author

Please use the space provided to explain your answers to the questions above. You may also include additional comments for the author, including concerns about dual publication, research ethics, or publication ethics. (Please upload your review as an attachment if it exceeds 20,000 characters)

Reviewer #1: The trial is well drafted but needed proactive understanding of the concept to enable drawing appropriate objectives. As described by the authors, there are several limitations of the study that impact the results obtained and conclusions. Further tests need to be done to understand the concept. Also, using susceptible mosquito population alone wasn’t ideal in situation where the spread of pyrethroid resistance represents a challenge for vector control. Knowing that the PIRK used is a transfluthrin-based product and also volatile. However, the study is a start point for improvement and could support outdoor biting vector control.

Few edits below

L33: authors should better highlight or refer directly to ITNs and IRS when talking about indoor control tools.

L52: should be An. gambiae s.l. and An. funestus s.l.

L60: consider both early biting and late morning biting

L87: remove “in mind” and maybe add “including entomological outcomes”

L145: can you specify the starving duration? It should be known and same for all replicates. That can be stated.

L165: It was stated earlier that the mosquitoes were released at 18:00. Here too, the collection start at 18:00. Is that means the collectors were seated before release? Or after. In any case, I think they should be a time to allow the mosquitoes acclimate in the chamber and be ready to seek for blood feeding by moving towards the collectors. Kindly check and clarify.

L194: it should have been interesting to continue to adulthood to check any male vs female difference. Because missing that, the fertility data lack some interpretations.

L251: The deterrence calculation seems confusing. The denominator of each ratio or percentage should be explained further. Considering that part of the mosquitoes was already collected by HLC collectors, can you explain how the denominators were estimated? If 7033/7974 were recollected by HLC in control chambers, it should remain 941 mosquitoes. Same for the PIRK chambers

L257: Same issues as above for the knock down too. Need to be clarified how the numbers were used. Between HLC collected and the calculation of KD

L274-275: Kindly harmonize the decimal digits to 1 throughout.

L295: I think it should be rather not significant

Reviewer #2: This manuscript is well written, well thought out and executed. I have made a few small editorial suggestions on the manuscript itself, but I have one point that must be covered in the discussion.

The authors correctly assert the importance of dealing with outdoor transmission, but the experiment is performed with a highly endophilic species. The authors comment on Aedes, but I think it must be noted that An. arabiensis is an important member of the An. gambiae complex that is far more exophilic and more likely to be involved in outdoor transmission. As such, this species is an ideal target for spatial repellents to deal with outdoor biting. This should be mentioned. Furthermore, if there is any further information on the effect of transfluthrin, a pyrethroid, on pyrethroid resistant species, this must either be mentioned in the discussion, or the gap acknowledged.

Otherwise, a well-executed paper.

**********

6. PLOS authors have the option to publish the peer review history of their article (what does this mean?). If published, this will include your full peer review and any attached files.

If you choose “no”, your identity will remain anonymous but your review may still be made public.

**Do you want your identity to be public for this peer review?** For information about this choice, including consent withdrawal, please see our Privacy Policy.

Reviewer #1: No

Reviewer #2: No

**********

[NOTE: If reviewer comments were submitted as an attachment file, they will be attached to this email and accessible via the submission site. Please log into your account, locate the manuscript record, and check for the action link "View Attachments". If this link does not appear, there are no attachment files.]

While revising your submission, please upload your figure files to the Preflight Analysis and Conversion Engine (PACE) digital diagnostic tool, https://pacev2.apexcovantage.com/. PACE helps ensure that figures meet PLOS requirements. To use PACE, you must first register as a user. Registration is free. Then, login and navigate to the UPLOAD tab, where you will find detailed instructions on how to use the tool. If you encounter any issues or have any questions when using PACE, please email PLOS at figures@plos.org. Please note that Supporting Information files do not need this step.

Attachment Submitted filename: PONE-D-22-32222_reviewer.pdf Click here for additional data file.

# Author comment: Author response to Decision Letter 0

**DOI:** 10.1371/journal.pone.0285501.r002

7 Apr 2023

Editor: 

Thank you for taking the time to review our manuscript, and for your flexibility in allowing us to extend our revision deadline. Individual comments are below:

1. I have adjusted the formatting of the manuscript to reflect the PLOS ONE style templates. 

2. The questionnaire is included. Most of the local approvals gained during this study were related to the second phase, a field study which is in preparation for submission, while this phase didn’t involve local community members.

3. The funders had no role in study design, data collection and analysis, decision to publish, or preparation of the manuscript.

4. I believe the copyrighted portion of Figure 1 is the floorplan of the semi-field chambers (Fig 1A). I took the opportunity to draft a new floorplan drawing which (if I understand correctly) should not require a copyright license agreement, and improves on the original figure by highlighting more relevant dimensions. The other two photographs are my own personal photographs and have not been published anywhere before.

5. I was not able to add the new references with tracked changes on due to my reference manager, but three additional references (#32-34) were added in the discussion in response to comments from reviewer #2 regarding the efficacy of transfluthrin against mosquitoes resistant to other pyrethroids.

Reviewer #1:

We would like to thank you for your time during your review of our manuscript. in response to your point about using a susceptible mosquito population alone, we agree that it is a limitation. We were unable to use a resistant strain since we didn’t have an available colony. We have added text to the manuscript to clarify that, and an additional paragraph in the discussion related to this and the similar limitation of using Anopheles gambiae when other species often exhibit the behaviors of interest (outdoor biting, etc.)

We agree and have incorporated each of your individual edits into the revised manuscript.

- Regarding starving duration, mosquitoes were starved for four hours. This has been clarified in the text. 

- The release and start times both being listed as 18:00 was a mistake that has been corrected. The mosquitoes were released in the chambers at 17:30 and the collectors would enter the chambers and start at 18:00. 

- The denominators in the text were a remnant from a previous draft where those numbers (e.g. 7974) had been discussed in the previous paragraph regarding recovery rates. The denominators referred to the total number of mosquitoes recovered from the experiments (excluding those that were missing/never recovered). They have since been amended, and the denominators now refer to the total number of mosquitoes released into the chambers. This also better reflects how the models were constructed, using the total release # as the offset in the Poisson models.

Reviewer #2:

We would like to thank you for the time taken during your consideration of our manuscript. We have incorporated your editorial suggestions into the revised manuscript and have included a paragraph in the discussion addressing your important points about An. arabiensis and the effect of transfluthrin on pyrethroid species. In brief, we agree regarding the behavior of the two (and other) vector species, and transfluthrin has been shown to inhibit pyrethroid-resistant species in the past, but there is no published data regarding resistance of mosquitoes towards transfluthrin or development of transfluthrin resistance in the field or lab.

Attachment Submitted filename: Response to Reviewers.docx Click here for additional data file.

# Peer review: Decision Letter 1

**DOI:** 10.1371/journal.pone.0285501.r003

**Authors:** Dziedzom Komi de Souza

25 Apr 2023

Semi-field evaluation of a volatile transfluthrin based intervention reveals efficacy as a spatial repellent and evidence of other modes of action

PONE-D-22-32222R1

Dear Dr. Burton,

We’re pleased to inform you that your manuscript has been judged scientifically suitable for publication and will be formally accepted for publication once it meets all outstanding technical requirements.

Within one week, you’ll receive an e-mail detailing the required amendments. When these have been addressed, you’ll receive a formal acceptance letter and your manuscript will be scheduled for publication.

An invoice for payment will follow shortly after the formal acceptance. To ensure an efficient process, please log into Editorial Manager at http://www.editorialmanager.com/pone/, click the 'Update My Information' link at the top of the page, and double check that your user information is up-to-date. If you have any billing related questions, please contact our Author Billing department directly at authorbilling@plos.org.

If your institution or institutions have a press office, please notify them about your upcoming paper to help maximize its impact. If they’ll be preparing press materials, please inform our press team as soon as possible -- no later than 48 hours after receiving the formal acceptance. Your manuscript will remain under strict press embargo until 2 pm Eastern Time on the date of publication. For more information, please contact onepress@plos.org.

Kind regards,

Dziedzom Komi de Souza, Ph.D.

Academic Editor

PLOS ONE

Additional Editor Comments (optional):

Reviewers' comments:

Reviewer's Responses to Questions

**Comments to the Author**

1. If the authors have adequately addressed your comments raised in a previous round of review and you feel that this manuscript is now acceptable for publication, you may indicate that here to bypass the “Comments to the Author” section, enter your conflict of interest statement in the “Confidential to Editor” section, and submit your "Accept" recommendation.

Reviewer #1: All comments have been addressed

Reviewer #2: All comments have been addressed

**********

2. Is the manuscript technically sound, and do the data support the conclusions?

The manuscript must describe a technically sound piece of scientific research with data that supports the conclusions. Experiments must have been conducted rigorously, with appropriate controls, replication, and sample sizes. The conclusions must be drawn appropriately based on the data presented. 

Reviewer #1: Yes

Reviewer #2: Yes

**********

3. Has the statistical analysis been performed appropriately and rigorously? 

Reviewer #1: Yes

Reviewer #2: Yes

**********

4. Have the authors made all data underlying the findings in their manuscript fully available?

The PLOS Data policy requires authors to make all data underlying the findings described in their manuscript fully available without restriction, with rare exception (please refer to the Data Availability Statement in the manuscript PDF file). The data should be provided as part of the manuscript or its supporting information, or deposited to a public repository. For example, in addition to summary statistics, the data points behind means, medians and variance measures should be available. If there are restrictions on publicly sharing data—e.g. participant privacy or use of data from a third party—those must be specified.

Reviewer #1: Yes

Reviewer #2: Yes

**********

5. Is the manuscript presented in an intelligible fashion and written in standard English?

PLOS ONE does not copyedit accepted manuscripts, so the language in submitted articles must be clear, correct, and unambiguous. Any typographical or grammatical errors should be corrected at revision, so please note any specific errors here.

Reviewer #1: Yes

Reviewer #2: Yes

**********

6. Review Comments to the Author

Please use the space provided to explain your answers to the questions above. You may also include additional comments for the author, including concerns about dual publication, research ethics, or publication ethics. (Please upload your review as an attachment if it exceeds 20,000 characters)

Reviewer #1: The authors have adequately addressed my comments. All others limitations of the study were already explained by the authors.

Reviewer #2: (No Response)

**********

7. PLOS authors have the option to publish the peer review history of their article (what does this mean?). If published, this will include your full peer review and any attached files.

If you choose “no”, your identity will remain anonymous but your review may still be made public.

**Do you want your identity to be public for this peer review?** For information about this choice, including consent withdrawal, please see our Privacy Policy.

Reviewer #1: No

Reviewer #2: No

**********

# Editor report: Acceptance letter

**DOI:** 10.1371/journal.pone.0285501.r004

**Authors:** Dziedzom Komi de Souza

2 May 2023

PONE-D-22-32222R1 

Semi-field evaluation of a volatile transfluthrin-based intervention reveals efficacy as a spatial repellent and evidence of other modes of action 

Dear Dr. Burton:

I'm pleased to inform you that your manuscript has been deemed suitable for publication in PLOS ONE. Congratulations! Your manuscript is now with our production department. 

If your institution or institutions have a press office, please let them know about your upcoming paper now to help maximize its impact. If they'll be preparing press materials, please inform our press team within the next 48 hours. Your manuscript will remain under strict press embargo until 2 pm Eastern Time on the date of publication. For more information please contact onepress@plos.org.

If we can help with anything else, please email us at plosone@plos.org. 

Thank you for submitting your work to PLOS ONE and supporting open access. 

Kind regards, 

PLOS ONE Editorial Office Staff

on behalf of

Associate professor Dziedzom Komi de Souza 

Academic Editor

PLOS ONE
